# Computational fragment-based drug design of potential Glo-I inhibitors

**DOI:** 10.1080/14756366.2024.2301758

**Published:** 2024-01-22

**Authors:** Roaa S. Bibars, Qosay A. Al-Balas

**Affiliations:** Department of Medicinal Chemistry and Pharmacognosy, Jordan University of Science and Technology, Irbid, Jordan

**Keywords:** Glyoxalase-I, fragment-based drug design, *de novo*, anti-cancer

## Abstract

In this study, a fragment-based drug design approach, particularly *de novo* drug design, was implemented utilising three different crystal structures in order to discover new privileged scaffolds against glyoxalase-I enzyme as anticancer agents. The fragments were evoluted to indicate potential inhibitors with high receptor affinities. The resulting compounds were served as a benchmark for choosing similar compounds from the ASINEX® database by applying different computational ligand-based drug design techniques. Afterwards, the selection of potential hits was further aided by various structure-based approaches. Then, 14 compounds were purchased, and tested *in vitro* against Glo-I enzyme. Of the tested 14 hits, the biological screening results showed humble activities where the percentage of Glo-I inhibition ranged from 0–18.70 %. Compound **19** and compound **28**, whose percentage of inhibitions are 18.70 and 15.80%, respectively, can be considered as hits that need further optimisation in order to be converted into lead-like compounds.

## GRAPHICAL ABSTRACT

**Figure:**
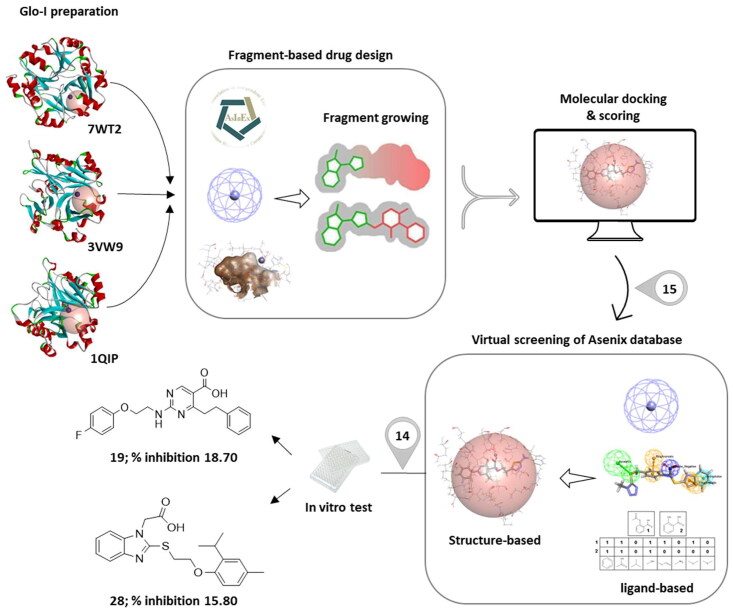

## Introduction

Cancer is a renegade growth system caused by the accumulation of genetic or epigenetic alterations in human body cells^1^^,^^2^. Despite the fierce battle being waged against cancer, it is still a significant health issue throughout the world^2^^,^^3^. According to World Health Organisation (WHO) statistics, cancer is the leading cause of death worldwide, accounting for 8.2 million deaths in 2012, and its prevalence is expected to grow to 13.0 million by 2030. Due to limited selectivity and lack of sufficient efficiency, the classical chemotherapeutic drugs have a substantial number of undesirable effects^1^. As a result, tremendous efforts have been conducted to discover the effective cure for cancer therapy, notably through identifying new reliable targets^4^. Recently, the glyoxalase system has drawn the attention of researchers as a potential target for the development of novel anticancer drugs^5^.

**Figure 1.**
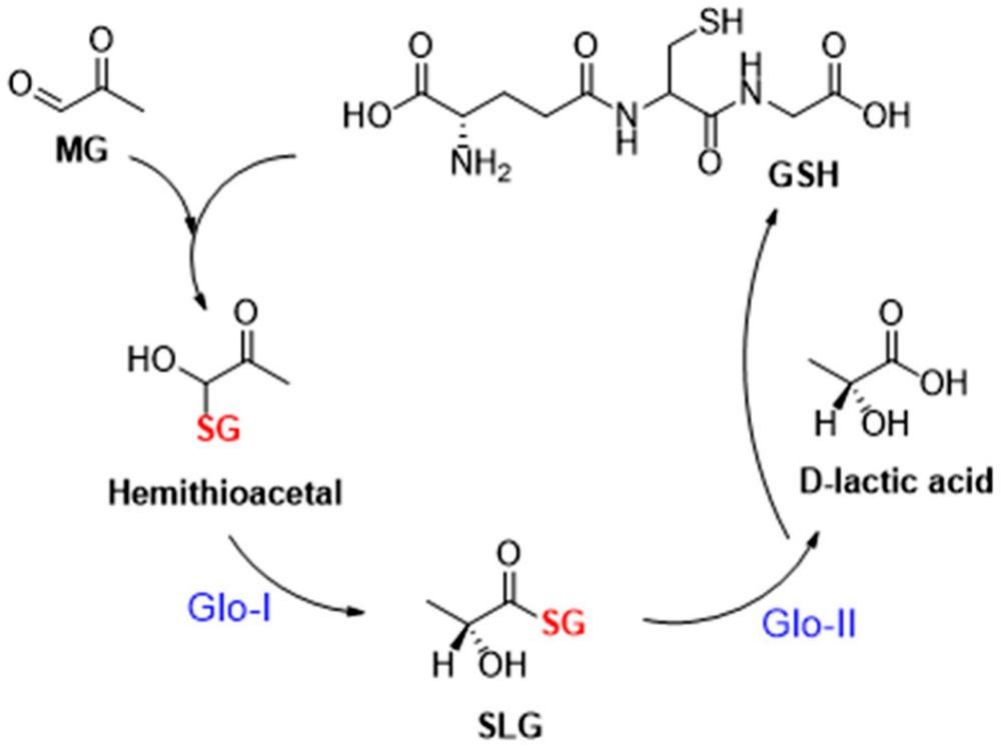
Glyoxalase cycle detoxification process.

The glyoxalase system is an enzyme-based detoxification system, consisting of two GSH-dependent enzymes; Glyoxalase-I and Glyoxalase-II that were discovered by Dakin and Dudley in 1913^6^,^7^. It plays a crucial function in the cellular metabolic pathways by converting the highly reactive, cytotoxic methylglyoxal into non-toxic lactic acid^7^^,^^8^. The methylglyoxal (MG) is a byproduct of non-enzymatic reactions with glyceraldehyde 3-phosphate (G3P) or dihydroxyacetone phosphate (DHAP)^9^^,^^10^. It is also a byproduct resulting from the metabolism of proteins, fatty acids, and glucose^9^. MG contributes to an increase in the oxidative stress due to inhibition of glutathione reductase^9^. Moreover, its cytotoxicity is attributed to its capacity to form proteins, lipids, and nucleic acids adducts, known as advanced glycation end-products (AGEs)^9^^,^^11^. These adducts are linked to a wide range of diseases, such as cardiovascular diseases^9^, Alzheimer’s disease^12^, depression^13^, anxiety^13^, aging^14^, diabetes^15^, obesity^9^, and cancer^9^^,^^16^^,^^17^. Hemithioacetal, which is produced non-enzymatically when methylglyoxal reacts with glutathione (GSH), is transformed by Glo-I enzyme into S, D-lactoylglutathione (SLG), which is then hydrolysed by Glo-II enzyme to produce D-lactic acid regenerating glutathione (Figure 1)^7^.

Malignant cells are known for their high metabolic rate and high glycolytic activity as observed by Warburgin in the 1920s^19^. The primary defence strategy to counteract toxic effects, implemented through increased glycolytic activity, is activation of detoxification systems^18^. Numerous cancer forms, including lung^19^, breast^11^, bladder^20^, and prostate^21^ exhibit overexpression of numerous detoxifying enzyme systems, particularly Glo-I. Moreover, Glo-I overexpression has also been connected to the multidrug resistance of many cancers, including prostate carcinoma^22^, lung carcinoma^19^, monocytic leukemia,^23^ and erythro leukaemia^23^. Consequently, it attracted particular interest in drug discovery as a potential target for combating cancer^23^. Therefore, utilising the discovery of Glo-I enzyme inhibitors as a therapeutic strategy may be beneficial in combating cancer-related pathological processes and reversing resistance to anticancer drugs caused by apoptosis. The development of approved Glo-I enzyme inhibitors over the past few decades has resulted in a variety of strengths, ranging from a high micromolar value to a low nanomolar IC_50_ or ki value^24^. These inhibitors are divided into two fundamental categories: GSH-based and non-GSH-based inhibitors.

**Figure 2.**
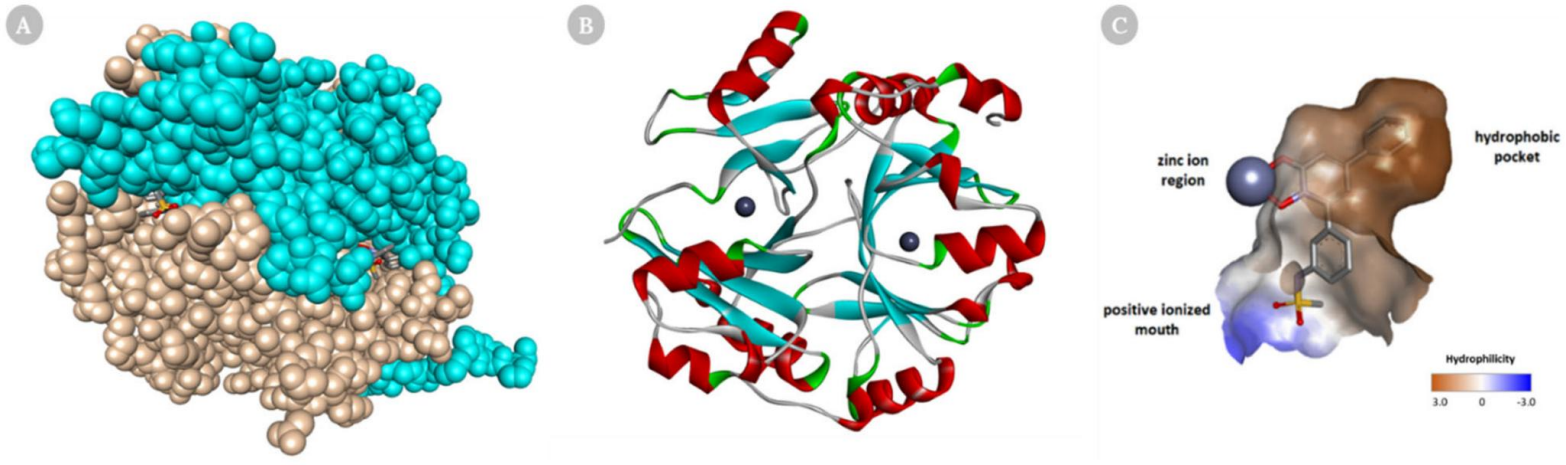
Cartoon representation of a 3D crystal structure of human Glo-I (PDB code 3W0T). (A) Surface representation, in which monomer A is coloured beige, and monomer B is coloured light blue. The co-crystalized inhibitors are shown in the stick representation (B) The protein is represented as solid ribbon, in which α-helix are in red, β-sheets are in cyan, turns are in green, loops are in white, and zinc metals are shown in dark grey sphere. (C) Three key areas of Glo-I active site: hydrophobic pocket (deep brown), positive ionised mouth (blue) and zinc ion region (the zinc atom is shown in dark grey sphere).

Glo-I is a homodimer, zinc metalloenzyme in which substrates and most inhibitors bind to the active site. Each monomer of Glo-I is made of 183 amino acids with molecular weight roughly 42 kDa. Structurally, Glo-I active site is located at the intersection of the two polypeptide chains and consists of three main regions for drug design: hydrophobic pocket, zinc ion region, and positive charge mouth. The Zn ion region, which coordinates with the amino acids Glu99, Gln33, Glu172, and His126 as well as one or two water molecules, mediates the catalytic transformation of Hemithioacetal. The positively charged mouth contains amino acids Arg37, Arg122, Lys150, and Lys156 making the entrance to the Glo-I active site very polar in nature. The water-impermeable hydrophobic pocket, which can hold up to two aromatic rings, is present deep inside the active site of Glo-I and contains amino acids Phe71, Phe62, Leu92, Leu69, Leu160, and Met179 (Figure 2)^24^.

In the present study, an extensive fragment-based drug design (FBDD) approach was utilised to unravel potential Glo-I inhibitors with promising activity as potential anticancer agents using three different 3D crystal structures of the human Glo-I enzyme available in the Protein Data Bank (PDB). This discipline deciphers the binding pockets inside the active site by measuring their potential energy to bind certain areas, then the top fragments observed bound in its target binding site are identified as anchor hits, which can then subsequently be advanced into a lead compound using a variety of optimisation approaches, which is referred to as fragment evolution. Various DS methods such as multiple copy simultaneous search (MCSS), CDocker, LibDock, ligand efficiency, and binding energy calculation were used to screen the designed ASINEX® fragment library in order to choose the core fragments that will eventually be evoluted into lead-like compounds. The evolved compounds were used as a guide to select similar compounds from the ASINEX^®^ commercial database. The similarity was based on both the 2D, and the 3D descriptors followed by performing extensive docking and energy binding calculations. Finally, the resulting compounds were purchased and evaluated biologically for their inhibitory activities against human Glo-I enzyme by *in vitro* assay.

## Results and discussion

### In silico drug design

#### Preparation of the Glyoxalase-I enzyme

Seven 3D solved crystal structures of the human Glo-I enzyme have been deposited in the PDB. The selection of appropriate Glo-I proteins was based on the following criteria: the nature of the co-crystallized ligand in terms of chemical structure, the resolution of the crystal, and the source of the papers that were published. The goal of the multiple selection was to investigate if the crystal structures have a high degree of similarity, and whether the resulting structures will also be roughly similar. Conversely, if the crystal structures are dissimilar, this will produce diverse compounds that cover a range of enzyme conformations. Therefore, three distinct crystal structures were chosen and employed as references to construct potential inhibitors, which are 7WT2, 3VW9, and 1QIP. The three crystal structures were prepared, solvated and energy minimised for further processing in three consecutive steps to gradually relax the protein models and remove any potential artefacts that could result from crystal packing as described in the method section.

**Scheme 1.**
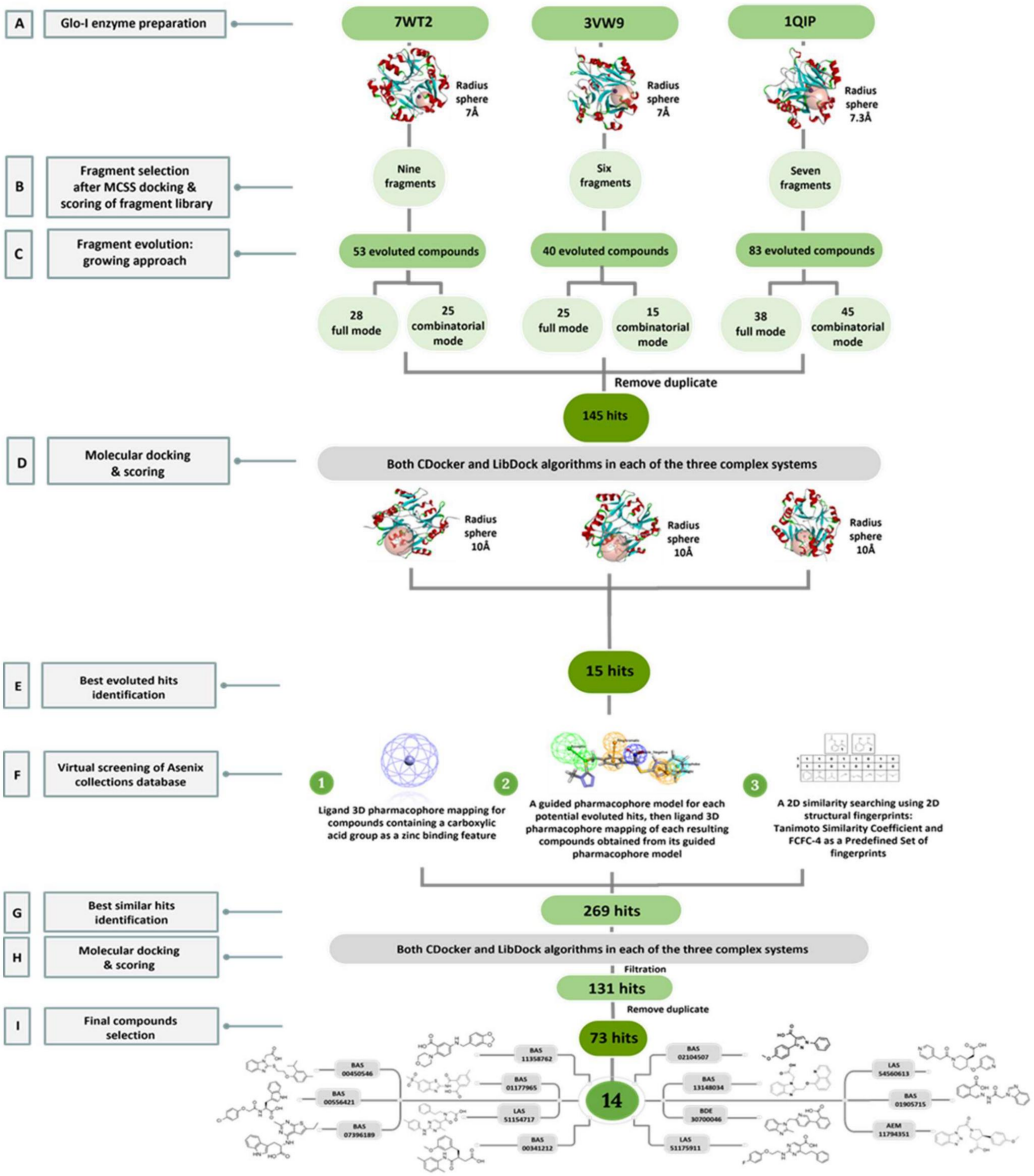
A flowchart summarising the computational results obtained regarding the identification of Glo-I inhibitors.

**Figure 3.**
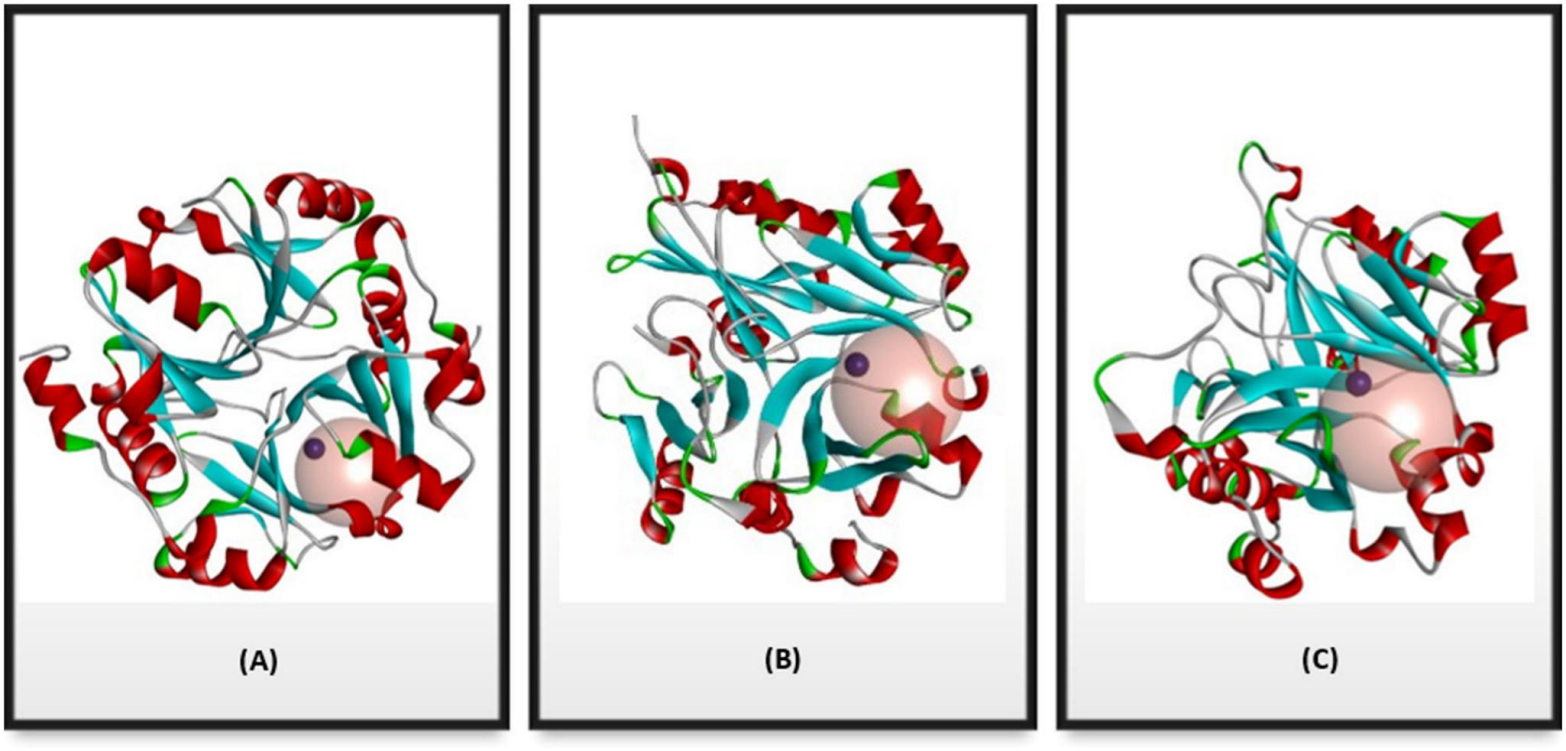
Glo-I PDB enzyme Preparation codes (A) 7WT2 (B) 3VW9 (C) 1QIP. The proteins are represented as solid ribbon, and the Zinc metals as dark red spheres. The active sites are defined with transparent spheres of (A) 7 Å (B) 7 Å (C) 7.3 Å radius.

Structurally, the Glo-I active site is divided into three main regions as mentioned previously. Regarding the FBDD approach that we applied, only two regions of the active site will be used which are the zinc ion region and a deep hydrophobic pocket. These two regions were defined into a single Zn-hydrophobic region because the zinc atom is remarkably close to the hydrophobic pocket. The binding site for each crystal structure of 7WT2 and 3VW9 was defined by a sphere of 7.0 Å radius, while the 1QIP crystal structure was defined by a sphere of 7.3 Å radius to ensure inclusion all crucial important amino acids of the two desired regions of the enzyme (Figure 3 and Scheme 1(A)).

#### Fragment library design

The commercially available ASINEX^®^ fragments library was downloaded and calculated the 2D descriptors, which will later be filtered and then employed in the following FBDD steps. The filtration was performed based on four criteria, referred to as the “Rule of Three”, namely molecular weight less than or equal to 300 Da, ALogP less than or equal to 3, hydrogen bond donors less than or equal to 3, and rotatable bonds to be less than or equal to 3. Despite the fact that the “Rule of Three” is universally acknowledged, different research teams’ interpretations have resulted in various guidelines^25^. In this study, the modification was related to hydrogen bond acceptors to be less than or equal to 6. The filtered step has retained 10,897 different fragments. Then, the retained library was converted into a 3D database and was ready for fragment screening step.

#### Primary screening of the fragment library

A virtual screening step of the 3D database was conducted prior to fragment docking, in order to expedite the process of fragments docking and to concentrate on those that may develop favourable interactions with the zinc-hydrophobic region. A search hypothesis comprises of 3D structure features that are desired to be detected in fragments database. In this study, a 3D customised pharmacophore zinc binding feature was utilised as a search query to identify fragments that possess zinc binding groups. This search retained 1,926 conformation fragment hits. The retained hits were then docked into the zinc-hydrophobic region.

#### Fragment docking and scoring

Generally, the computational docking algorithms for FBDD within DS are classified into two major groups which are De Novo-based protocols^26^ and CHARMm-based MCSS protocol^27^. Based on the nature of the active site of the protein target, the nature of the potential ligand, the type of effective interactions that are formed with this potential ligand, and the specific goals of the study can be guide the selection of the best appropriate approach to be employed in a FBDD study. At this stage of the study, the focus on the Glo-I enzyme was related to the central zinc ion, the hydrophobic pocket, and the crucial metal acceptor interaction that a fragment should establish within the active site in order to demonstrate high binding affinity. The retained hits from the virtual screening step were then docked into the Zn-hydrophobic sphere using MCSS protocol as it concentrates on calculating electrostatic interactions rather than hydrogen bonds and hydrophobic interactions as Ludi-based protocol does. The MCSS docking protocol involves the simultaneous searching of multiple copies of a fragment, each with a different orientation and conformation, against the target protein, allowing for a more efficient exploration of conformational space and better analysing of the binding pocket. CHARMm force field minimisation is performed to define energetically favourable fragment positions. The placed fragments are ranked according to energy function by MCSS score. The MCSS score is a negative energy value of the total interaction energies of the fragment-protein interaction energy and the fragment energy.

The identification of 1,462, 718, and 873 different conformations in the crystal structures of 7WT2, 3VW9, and 1QIP, respectively, was achieved by a threshold cut off set of 80 or greater for the MCSS score of the retained 1,926 fragments for further investigation via calculating their free binding energy and ligand efficiency. The coordination geometries of the zinc atoms, which are square pyramidal in both the 7WT2 and 1QIP structures and tetrahedral in the 3VW9 structure, may explain the inconsistent results between the protein crystal structures. In addition, the properties of the amino acids located within the defined sphere of each structure and the nature of the co-crystallized ligand within the active site could also play a key role.

#### Calculation of the total binding energy and ligand efficiency of the filtered docked fragments

The total binding energies of the filtered fragments retained from docking into the Zn-hydrophobic region were calculated using the In Situ Ligand Minimisation and the Calculate Binding energies protocols. The In Situ minimisation step is performed prior to binding energy calculation to optimise the ligand in the binding pocket. The calculate binding energy step is performed to take into consideration ΔG of binding or solvation/desolvation energy and entropy^28^. It estimates binding free energy using CHARMm implicit solvation models, where the free binding energies were calculated from the free energies of the complex, the receptor, and the ligand using the following equation:

$${\text{Energy}}_{\text{Binding}}={\text{Energy}}_{\text{Complex}}–{\text{ Energy}}_{\text{Ligand}}–{\text{ Energy}}_{\text{Receptor}}$$

The Poisson Boltzmann with non-polar surface area (MM-PBSA) model was selected because it is the most rigorous implicit solvent model^29^, while the ligand conformation entropy was set to true to be able to calculate the total binding energy, which is the sum of binding energy and ligand conformation energy, which represents the energy difference of the ligand to lowest energy conformation.

Moreover, the ligand efficiency of docked fragments was also calculated, which is used to evaluate the binding affinity of small fragments to a target protein. It attempts to normalise a fragment’s activity by its molecular size; as a result, it serves as an indirect indicator of the number of its constituent atoms interacting with the target protein^30^. A negative LE value is advantageous since docked fragments estimated binding energies are indicated to have a negative value. It is calculated from the binding affinity of the molecule and its molecular weight using the following equation:

$$\text{LE}=-\text{RTln}\left({\text{IC}}_{50}\right)/\text{Molecular weight}$$

The total free binding energies of the filtered fragments were ranged from −58.1908 to 36.4152, −40.9643 to 52.5446, and −25.1623 to 35.3625 in the crystal structures of 7WT2, 3VW9, and 1QIP, respectively, and their ligand efficiency values were ranged from −0.25014 to 0.20894, −0.209834 to 0.26287, and −0.15122 to 0.17184 in the same order of the crystal structures as above.

#### Selection of the core fragments

**Figure 4.**
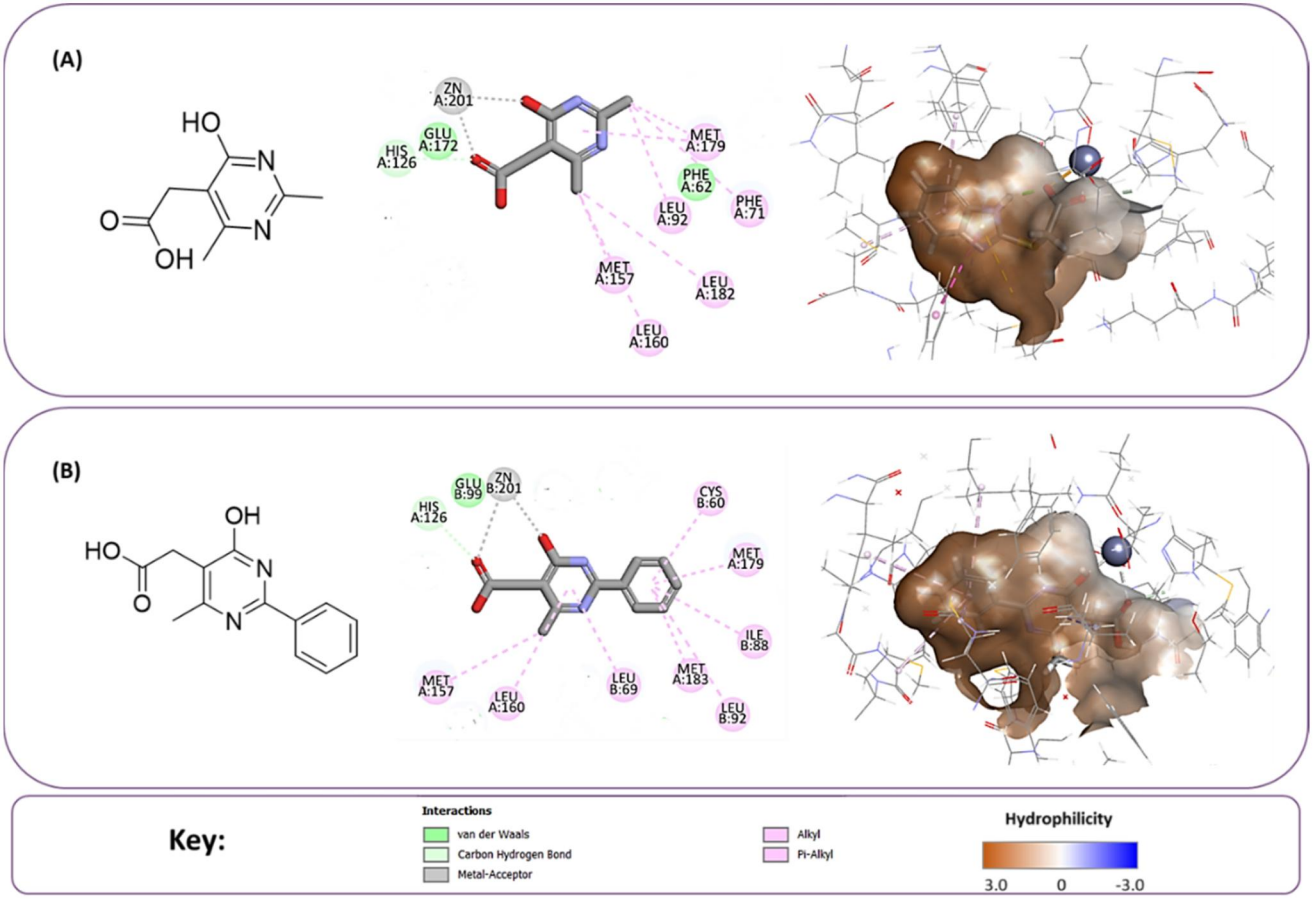
The docked poses of fragments binding the Zn-hydrophobic region of (A) fragment **A** and (B) fragment **B**, in the active site of Glo-I PBD code (A) 7WT2 (B) 3VW9 along with a 2D interaction map using MCSS. The binding site is represented as a hydrophobic surface, and the Zinc metal as a grey sphere.

Further inspection and filtration were conducted to select the most suitable fragments, which will be used to construct the final lead compounds. The alignment of the docked poses was visually inspected, and then the fragments were chosen based on a variety of factors, such as the coexistence of the desired hydrophobic functionalities and zinc coordination groups, the formation of favourable interactions by the fragments, the MCSS docking score, the binding interaction energy, and the ligand efficiency. For instance, when the Zn^+2^ metal interacts with a negatively ionising functional group, particularly a carboxylic acid, through a coordination interaction, or when the hydrophobic functionalities are occupying the hydrophobic pocket (Figure 4). Based on these criteria, nine core fragments fitting the Zn-hydrophobic region were selected in the 7WT2 structure, six in the 3VW9 structure, and seven in the 1QIP structure .In all three systems, the selected fragments were identical. Three fragments in the 3VW9 structure and two fragments in the 1QIP structure were previously excluded since their MCSS docking values dropped below the cut-off threshold (Table 1 and Scheme 1B).

**Table 1.** The selected core fragments binding the Zn-hydrophobic region with their scores in the three crystal structures.

Ind ex	Chemical Structure	7WT2			3VW9			1QIP			M.W.
		MCSS Score	TBE^a^	LE^b^	MCSS Score	TBE^a^	LE^b^	MCSS Score	TBE^a^	LE^b^	
**A**	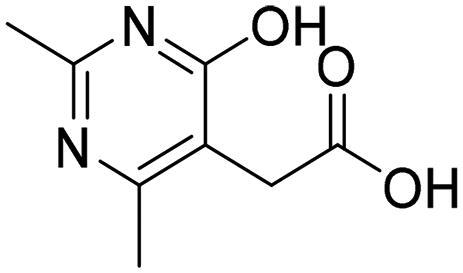	128.924	−38.5808	−0.2118	104.439	16.0013	0.0878	110.769	3.6113	0.0198	182.177
**B**	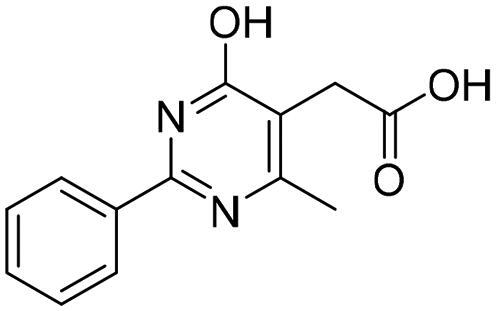	119.155	−43.8544	−0.1796	93.551	−21.0925	−0.0864	99.850	5.0888	0.0208	244.246
**C**	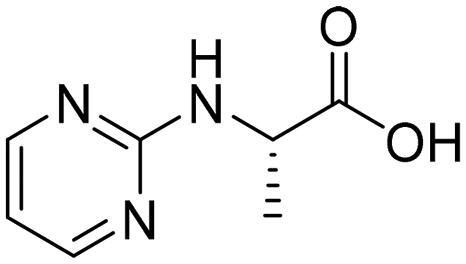	114.736	−23.3095	−0.1394	106.148	7.6671	0.0459	110.368	8.3500	0.0499	167.165
**D**	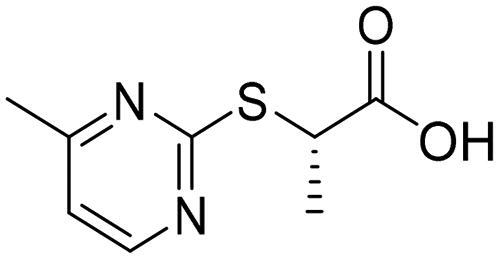	96.0731	−15.6493	−0.0790	92.277	6.0845	0.0307	91.429	−10.9028	−0.0549	198.242
**E**	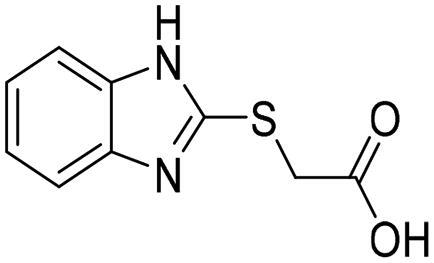	95.155	6.0724	0.0293	84.024	21.4516	0.1035	84.455	9.0079	0.0435	207.229
**F**	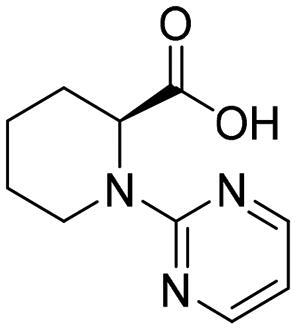	93.834	−16.9692	−0.0819	90.962	−3.8220	−0.0184	89.419	−13.1670	−0.0635	207.229
**G**	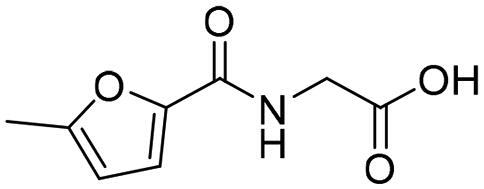	89.374	−8.2936	−0.0453	*	*	*	80.582	10.4577	0.0571	183.161
**H**	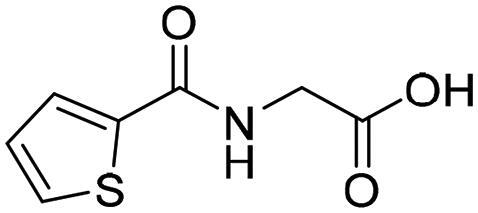	85.792	−19.2547	−0.1040	*	*	*	*	*	*	185.200
**I**	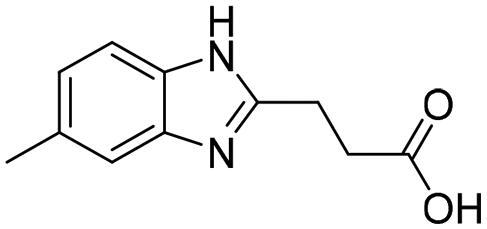	81.840	−18.0054	−0.0882	*	*	*	*	*	*	204.225

^a^TBE: Total binding energy in Kcal/mol, ^b^LE: Ligand efficiency

* Indicates that the MCSS score was less than the cut-off value

#### Fragments evolution

The final selected core fragments were evoluted towards generation of the final compounds using the De Novo Evolution protocol. This protocol generates full compounds in the binding site of the receptor based on an initial fragment. A collection of compounds with higher scores is created by covalently fusing fit fragments to the core fragment in a complementary way to the active site utilising the Ludi program^31^. The nature of the fragment selection and construction of new compounds depends on the evolution mode, with three distinct modes available in DS: full, quick, and combinatorial evolution.

In this study, the evolution step was performed using two modes, namely full evolution, and combinatorial evolution modes in the three complex systems. The full evolution mode in which the fragments are fused to the core fragment in an evolutionary fashion and the survivors are selected from generations of compounds ranked by a scoring function for the next iteration of fusing. Whereas in the combinatorial evolution mode, all fitted fragments are enumerated at the specified link sites in the core fragment. The Glo-I TLSC702 reveal inhibitor (5ZO) complex was utilised to define the binding site of the 7WT2 crystal structure; the Glo-I N-hydroxypyridone derivative inhibitor (HPJ) complex to define the binding site of the 3VW9 crystal structure; the Glo-I S-P-nitrobenzyloxycarbonyl glutathione inhibitor (GNB) complex to define the binding site of the 1QIP crystal structure, and then performed the full evolution mode of the selected core fragments in each of the three complex systems.

Toxic functional groups and compounds with more than ionisable groups were removed from the final compounds obtained from both evolution modes in each of the three systems. Subsequently, the duplicated compounds were removed from three complex systems and the two modes of evolution, resulting in the identification of 145 unique hits, which were chosen for molecular docking (Scheme 1C). As a consequence, there is a significant degree of similarity between the crystal structures; 20–25% of the resulting structures were identical, while the remainder was largely comparable.

#### Molecular docking of evoluted compounds and scoring

In order to assess the efficiency and accuracy of the docking algorithm in pose prediction, the co-crystallized ligand for the three complex system was retrieved from the complex and redocked into its defined Glo-I active site. Once the co-crystallized and redocked ligand pose are aligned, the docking algorithm can proceed for the evoluted compounds. The heavy-atom RMSD values were calculated for the native co-crystallized ligands (5ZO, HPJ, and GNB) and the corresponding redocked ligands using CDocker. In the crystal structures of 7WT2, 3VW9, and 1QIP, the RMSD values for the ligands were 0.4694, 1.6248, and 1.2690, respectively. Similarly, when employing LibDock, the RMSD values for the ligands were 0.3099, 0.5318, and 1.5257 in the same sequence as mentioned earlier.

Following the validation step, the prepared 145 hits were docked into the defined active site using CDocker and LibDock algorithms, as presented in the methods section. In DS, a grid-based molecular dynamic docking method called CDocker was employed to execute molecular docking on the retained hits. Elevated temperature molecular dynamics is used in this approach, then random rotations, to account for full ligand flexibility while treating the protein as a rigid molecule. To further refine the docked poses, the algorithm then executes an additional phase of simulated annealing or minimisation. The created poses were then graded based on CDocker energy, which was determined based on the internal ligand strain energy and the receptor-ligand interaction energy, and CDocker interaction energy, which was determined based on the non-bonded interaction energy only that exists between the protein and ligand. At both energy grades, the larger -CDocker interaction energy and -CDocker energy indicate a very favourable interaction between the protein and the ligand^32^. The calculated -CDocker interaction energy scores of the top scoring pose of the docked evoluted compounds ranged from 39.6745 to 61.4423 kcal/mol, 35.6736 to 62.2188 kcal/mol, and 42.5957 to 76.0942 kcal/mol in the crystal structures of 7WT2, 3VW9, and 1QIP, respectively. Whereas LibDock is a site-feature docking algorithm developed by Diller and Merz that docks ligands into an active site under the guidance of binding hotspots. This docking drive considers the flexibility of the ligand while treating the receptor as a rigid molecule^33^. The calculated LibDock scores of the top scoring pose of the docked evoluted compounds ranged from 84.933 to 151.946 kcal/mol, 80.274 to 141.137 kcal/mol, and 86.687 to 148.355 kcal/mol in the crystal structures of 7WT2, 3VW9, and 1QIP, respectively.

Moreover, a more robust computational method available in DS was used to further estimation of the free binding energies of the evoluted compounds for both CDocker and LibDock docked ligands using Calculating Binding Energy protocol with MM-PBSA implicit solvent model. The calculated total binding energy scores of the best scoring pose of the CDocker docked compounds ranged from 30.7203 to −41.3385 kcal/mol, 18.7497 to −65.4105 kcal/mol, and 12.6023 to −73.4002 kcal/mol in the crystal structures of 7WT2, 3VW9, and 1QIP, respectively, and 16.8233 to −54.8923 kcal/mol, −6.3235 to −66.7446 kcal/mol, and −5.7062 to −89.0642 kcal/mol for the best scoring pose of the LibDock docked compounds in the same order of the crystal structures as above (Scheme 1D).

#### Selection of the evoluted compounds

**Figure 5.**
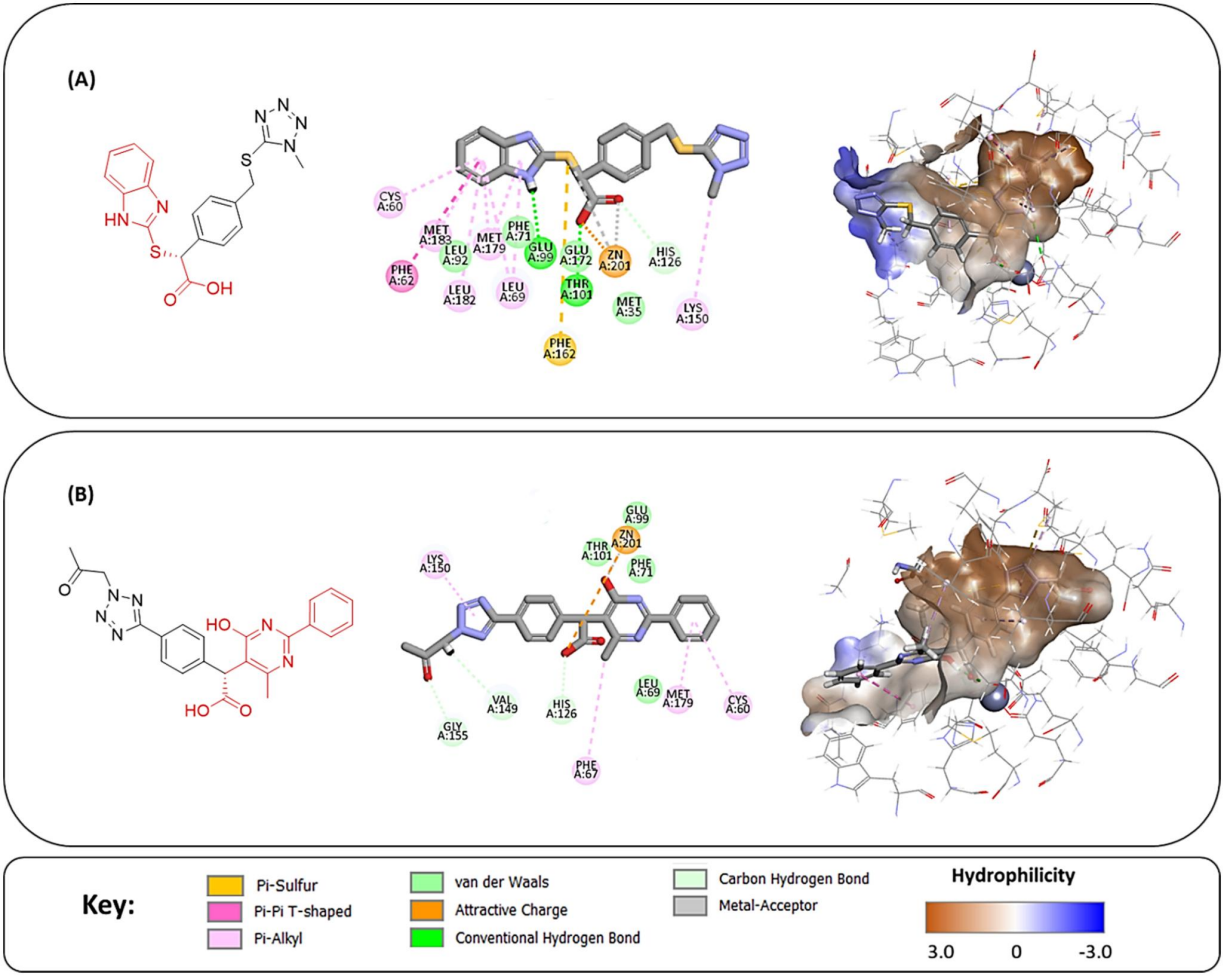
(A) The docked pose of full evoluted mode of compound number **1** in the active site of Glo-I (PBD code 7WT2) along with a 2D interaction map using CDocker. (B) The docked pose of combinatorial evoluted mode of compound number **5** in the active site of Glo-I (PBD code 3VW9) along with a 2D interaction map using CDocker. The binding site is represented as a hydrophobic surface, and the Zinc metal as a grey sphere.

**Figure 6.**
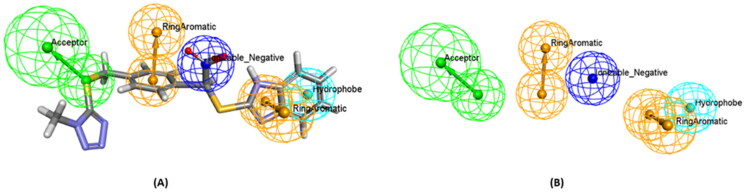
(A) A guided pharmacophore model generated from compound number **1** (B) The compound has been eliminated for clarification. The feature types in this pharmacophore are: HBA (green), NI (dark blue), HY (sky blue), and RA (brown).

Of the 145 docked hits, 15 hits were selected based on all scored energy parameters, favourable 2D interactions, and polarity. In full evolution mode, fragment number **B** was evoluted and let to generation the final compounds number **4**, **9**, and **10**, fragment number **C** generated the final compounds number **12** and **13**, fragment number **D** generated the final compound number **15**, and fragment number **E** generated the final compounds number **1** (compound 1 in Figure 5A), **2**, **3**, and **8**. Whereas, in combinatorial evolution mode, fragment number **D** was evoluted to generated the final compound number **11**, fragment number **F** generated the final compound number **14**, fragment number **G** generated the final compound number **7**, and fragment number **I** generated the final compounds number **5** (compound 5 in Figure 5B) and **6**. However, the evoluted compounds that emerged from both fragment **A** and **H** were excluded because they generated high polar compounds, which was unfavourable property in our research. Those 15 final hits were chosen as potential inhibitors of the Glo-I enzyme (Table 2 and Scheme 1E).

**Table 2.** The 15 selected evoluted final compounds with their top scores in the 7WT2, 3VW9, and 1QIP crystal structures.

Index	Chemical Structure	Glo- I Enzyme	CDocker		Molecular Weight	LibDock		ALog P
			-CDocker Interaction Energy	Total Binding Energy		LibDock Score	Total Binding Energy	
**1**	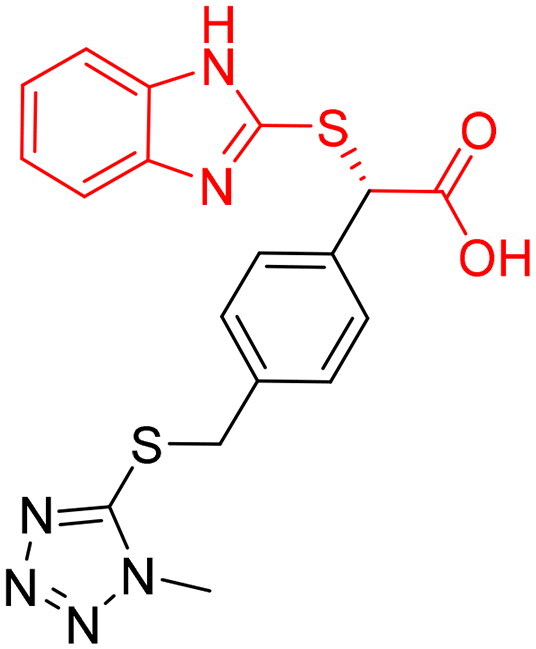	**7WT2**	50.3684	−7.0470		117.609	−23.3537	
		**3VW9**	47.1596	−34.2636	411.480	115.302	−37.4981	2.132
		**1QIP**	58.5484	−56.9211		130.031	−52.1568	
**2**	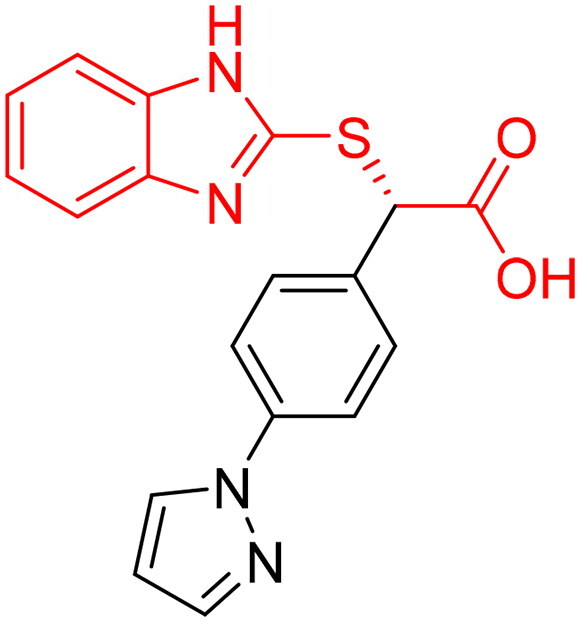	**7WT2**	47.0887	−11.8196		109.574	−16.2248	
		**3VW9**	43.1271	−16.7558	349.390	104.293	−27.9846	2.381
		**1QIP**	53.8044	−46.2009		109.645	−37.8752	
**3**	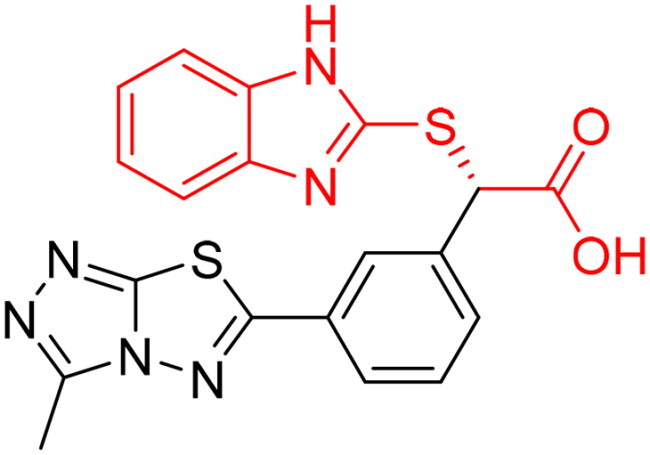	**7WT2**	52.4575	−8.5754		116.601	−28.4698	
		**3VW9**	48.2435	−30.2753	421.470	114.114	−34.3331	2.039
		**1QIP**	54.5566	−11.3702		121.942	−57.4170	
**4**	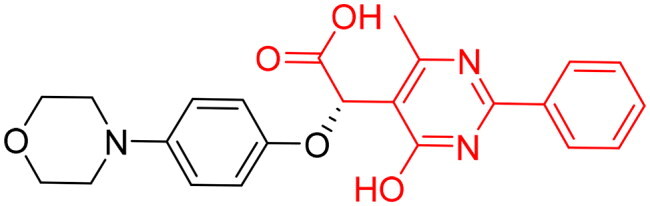	**7WT2**	56.5410	−26.1861		129.359	−34.0136	
		**3VW9**	48.2608	−43.9945	421.450	120.940	−38.9095	1.810
		**1QIP**	59.7007	−60.3470		135.606	−59.4032	
**5**	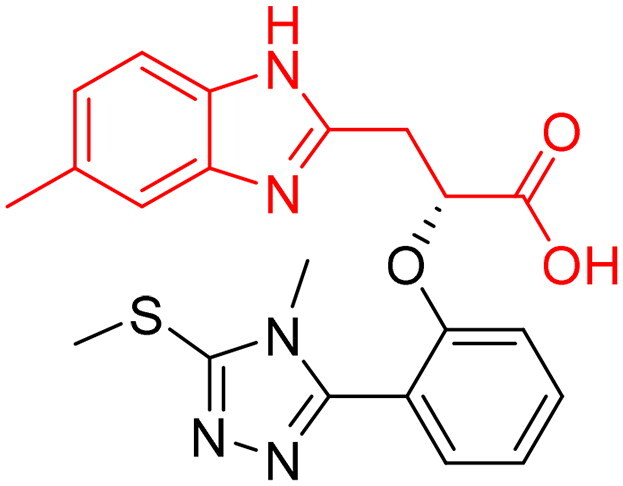	**7WT2**	54.0833	−30.9362		116.431	−34.6390	
		**3VW9**	48.3703	−26.7254	423.488	114.014	−51.5502	2.561
		**1QIP**	53.9563	−46.3827		114.272	−59.4233	
**6**	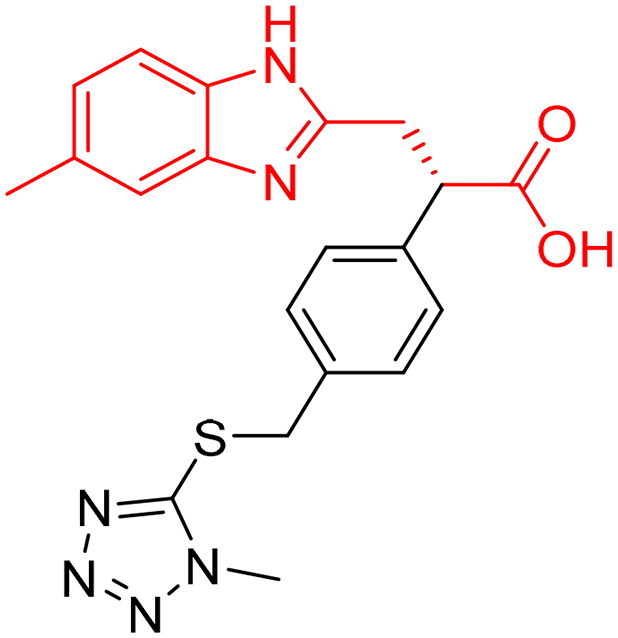	**7WT2**	53.5941	−14.1825		123.002	−33.2717	
		**3VW9**	46.0185	−52.0834	408.477	118.842	−27.7652	1.935
		**1QIP**	56.6467	−58.0371		130.763	−60.2848	
**7**	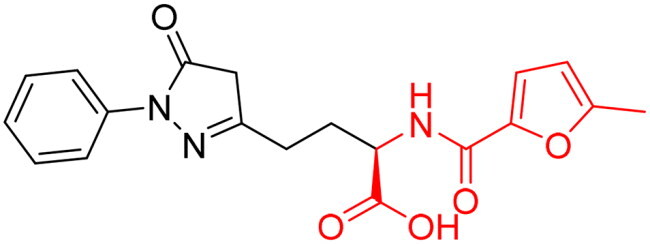	**7WT2**	54.5461	−16.2725		113.595	−47.7952	
		**3VW9**	49.8029	−42.2734	369.371	108.521	−38.3520	0.100
		**1QIP**	62.4412	−44.0861		134.995	−60.1828	
**8**	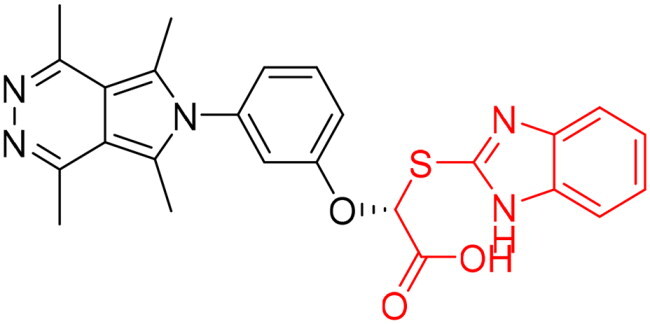	**7WT2**	45.1253	10.8782		123.309	−41.2757	
		**3VW9**	52.0187	−16.5936	472.540	124.925	−43.7194	3.509
		**1QIP**	53.9657	−44.0861		134.995	−72.6510	
**9**	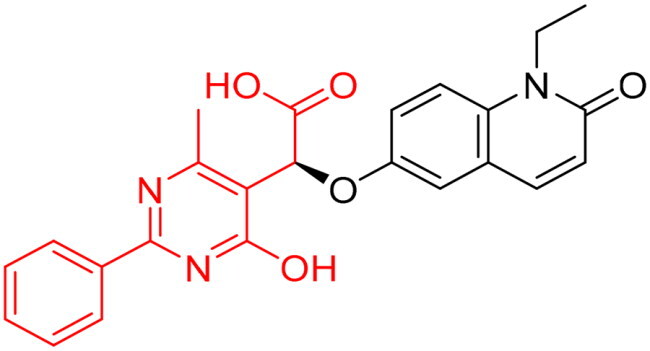	**7WT2**	54.1659	−10.3910		136.546	−24.5396	
		**3VW9**	49.1098	−27.2910	431.450	141.137	−44.3737	1.938
		**1QIP**	55.8129	−60.2227		138.725	−48.2928	
**10**	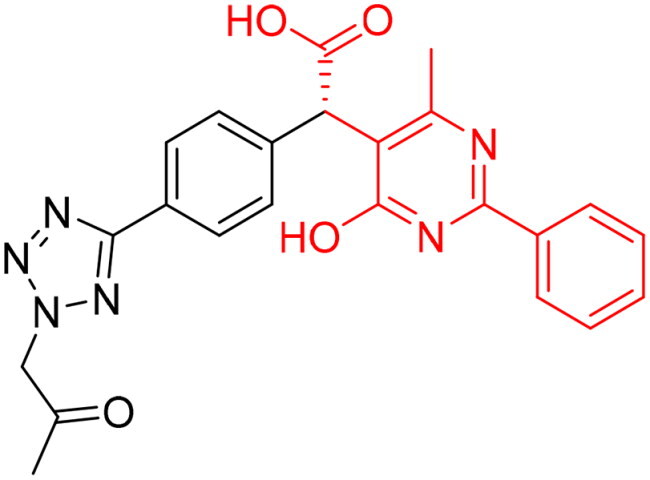	**7WT2**	56.0225	−18.5068		141.603	−32.4835	
		**3VW9**	45.3146	−32.2022	444.450	89.119	−31.2570	1.193
		**1QIP**	63.8028	−64.1539		145.478	−72.6252	
**11**	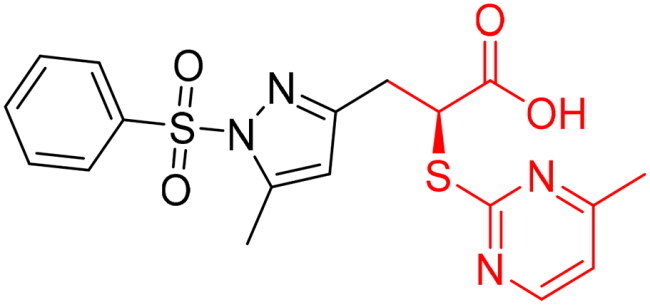	**7WT2**	50.5520	−13.5929		119.166	−20.2099	
		**3VW9**	51.6124	−18.4359	418.490	115.219	−37.4601	1.533
		**1QIP**	58.8910	−41.2374		117.273	−41.8113	
**12**	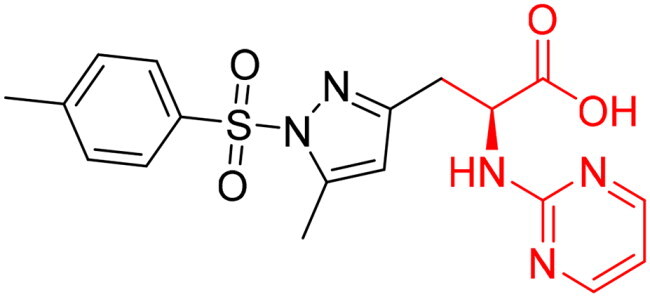	**7WT2**	49.9588	7.4335		114.227	−19.6695	
		**3VW9**	50.8205	−16.0488	401.440	113.881	−33.1467	1.003
		**1QIP**	64.1854	−36.4956		117.947	−44.7473	
**13**	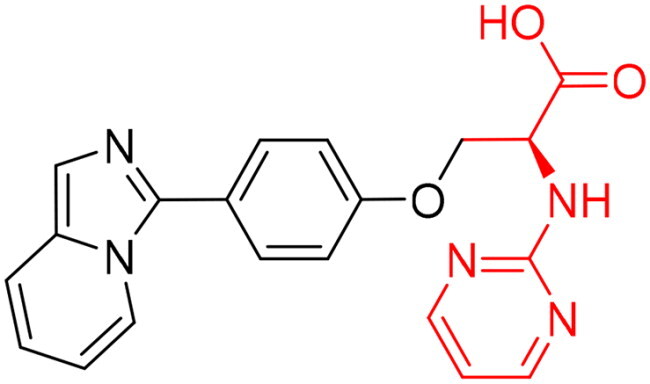	**7WT2**	48.8439	6.2066		121.848	−42.6983	
		**3VW9**	51.5975	−57.0814	375.390	111.879	−30.4650	0.843
		**1QIP**	58.8511	−48.8392		118.922	−60.2968	
**14**	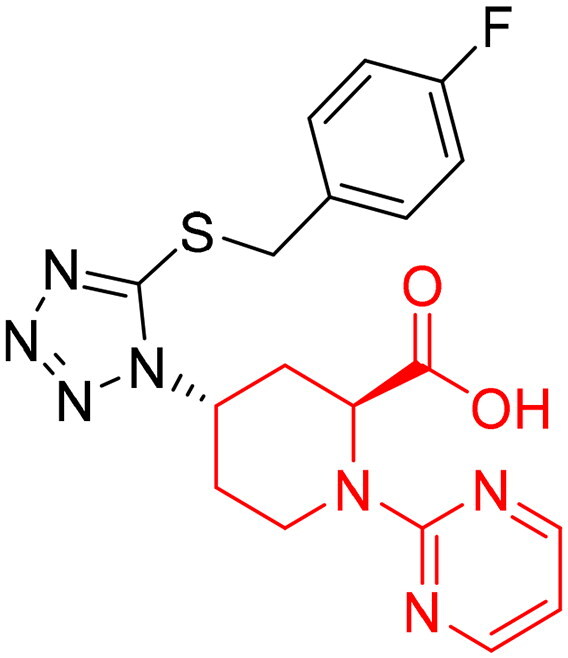	**7WT2**	48.8895	−13.9340		113.217	−27.2947	
		**3VW9**	45.7723	−38.6512	415.445	116.987	−40.1945	0.680
		**1QIP**	56.7063	−50.0110		119.567	−59.6618	
**15**	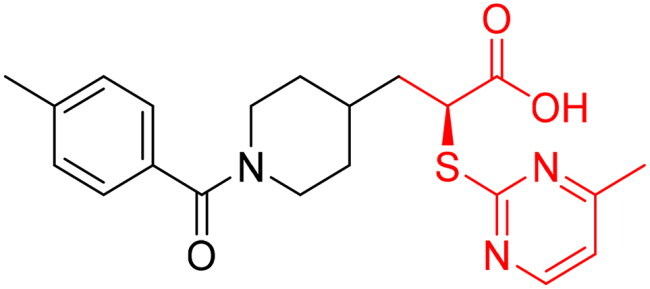	**7WT2**	52.7207	−26.9527		115.168	−30.7594	
		**3VW9**	50.8553	−38.9541	399.510	112.012	−48.8994	2.027
		**1QIP**	64.7172	−73.4002		114.758	−62.0532	

#### Virtual screening of commercial database

To assess the worthiness of synthesising evoluted compounds as the evolution approach has developed sophisticated and optimised compounds that possessing unique functionalities, exploration of similar compounds before embarking on a synthetic endeavour has become critical. Due to the fact that similar compounds virtually possess the same biological activity and features, it is possible to gain valuable insights into the potential benefits and drawbacks of synthesising evoluted compounds. This strategy encourages effectiveness, enhances functionality and assessment, and guarantees that resources are allocated towards compounds that have the greatest potential for scientific advancement and usability.

The ASINEX® screening collections database, which contains more than 500,000 compounds with prospective diversity metrics, was screened for potential hits. Several approaches were used to search for similar compounds in virtual screening in accordance with ligand-based screening method including, pharmacophore-based and fingerprint-based similarities. Both rely on the similarity between the 2D or 3D substructures of known ligands and the reference molecule and search a database for compounds that fit the query^34^. The final evoluted compounds were utilised as a guide for our selection of similar compounds.

Firstly, a 3D customised pharmacophore zinc binding feature assigned to a carboxylic acid group only was utilised as a search query to concentrate on those that may develop favourable interactions with the zinc region and to identify compounds that possess a zinc binding group as the evoluted compounds. This search retained 14,839 conformation hits. Subsequently, a guided ligand-based pharmacophore model was developed from each of the evoluted compounds to obtain as similar compounds as possible in terms of the shape, position of the features and similar interactions between the ligand-receptor complex using Ligand Pharmacophore Mapping protocol (Figure 6 and Scheme 1F) (Different guided pharmacophore models and their validation are presented in Supplementary 1). Mapping compounds generated from each evoluted compound were used as input ligand in Find Similar Molecules by Fingerprints protocol. These approaches resulted in the selection of 269 compounds from all reference ligands (Scheme 1G).

#### Selection of the final compounds

Molecular docking of the 269 selected hits was performed using CDocker and LibDock protocols in each of three systems. Further visual inspection and filtration were conducted to select the most suitable similar compounds based on a variety of factors, such as the alignment of the docked poses, the coexistence of the desired hydrophobic functionalities, the formation of favourable interactions by the compounds, and the docking scores. This filtration led to the identification of 131 compounds and then the removal of the duplicates led to the identification of 73 different hits (Scheme 1H and 1I).

**Figure 7.**
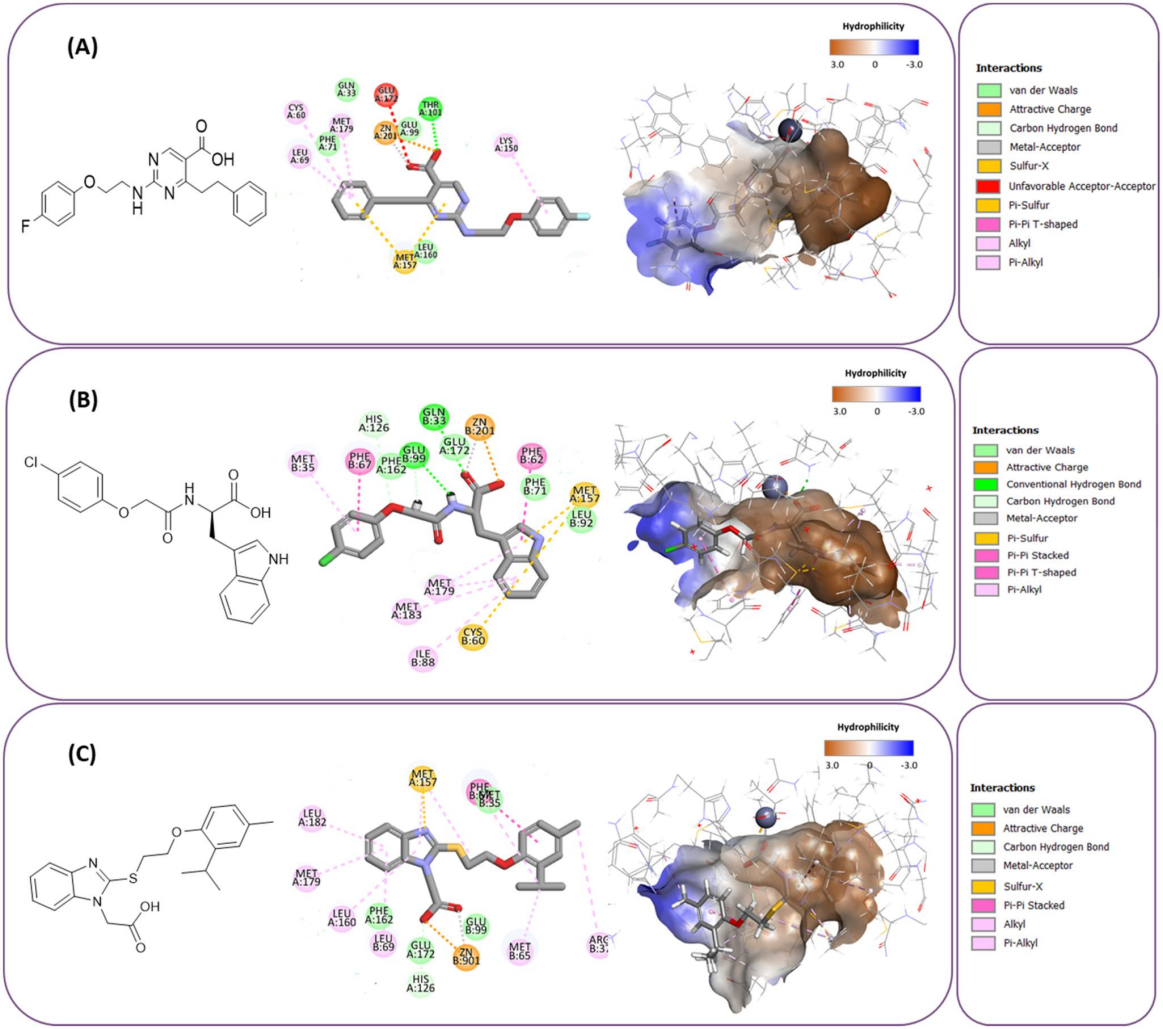
The docked pose of final compounds of (A) compound number **19** (B) compound number **24** (C) compound number **28**, in the active site of Glo-I PBD code (A) 7WT2 (B) 3VW9 (C) 1QIP along with a 2D interaction map using CDocker. The binding site is represented as a hydrophobic surface, and the Zinc metal as a grey sphere.

To identify the most potential inhibitors of the Glo-I enzyme, additional investigation and filtering procedures were conducted. The optimal compounds were selected by considering numerous factors, including docking scores, binding energy, total binding energy, polarity, molecular weight, and forming good binding interactions with the binding site especially with zinc ion and the hydrophobic pocket. Moreover, the Cluster Ligands protocol was utilised to assist in the final selection and ensure the diversity of the chosen compounds. These criteria allowed for the identification of 14 compounds, which were purchased and evaluated for activity according to the developed biological assay in our research lab (Figure 7 and Table 3). A summary of the entire computational results obtained regarding the identification of Glo-I inhibitors in this project is illustrated in Scheme 1.

**Table 3.** The 14 selected final similar compounds with their top scores in the 7WT2, 3VW9, and 1QIP crystal structures.

Index	Chemical Structure and IDNUMBER	Glo-1 Enzyme	CDocker			M.W.	LibDock			Alog P
			-CDocker Interaction Score	Binding Energy	Total Binding Energy		LibDock Score	Binding Energy	Total Binding Energy	
**16**	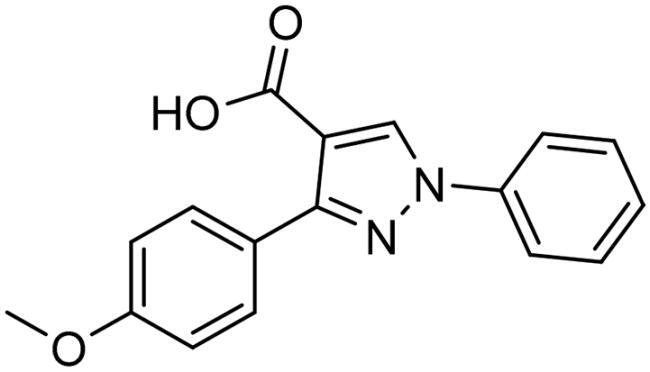 BAS 02104507	**7WT2**	44.2703	3.0430	4.2749		78.082	−41.6635	−40.4316	
		**3VW9**	44.1337	−14.2352	−13.0033	293.297	80.182	−33.0722	−31.8403	2.142
		**1QIP**	49.6386	−48.5148	−46.8301		77.002	−23.2622	−22.0303	
**17**	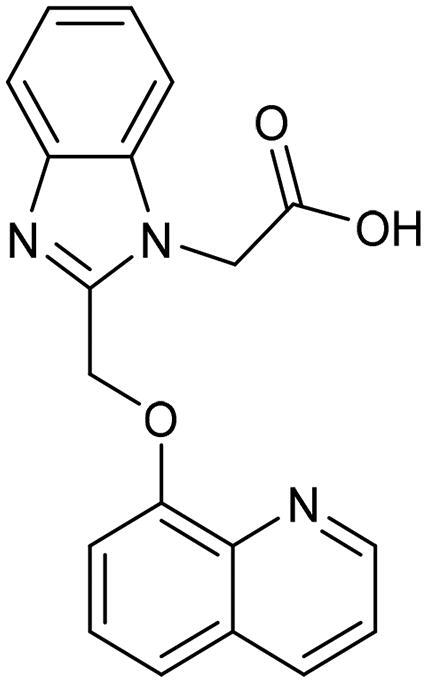 BAS 13148034	**7WT2**	47.3871	−2.6470	−1.0419		110.998	−28.2252	−20.9951	
		**3VW9**	46.4587	−47.1887	−44.5539	332.333	108.183	−43.0474	−37.7114	1.505
		**1QIP**	54.8069	−52.7042	−50.0743		110.543	−35.6077	−34.5216	
**18**	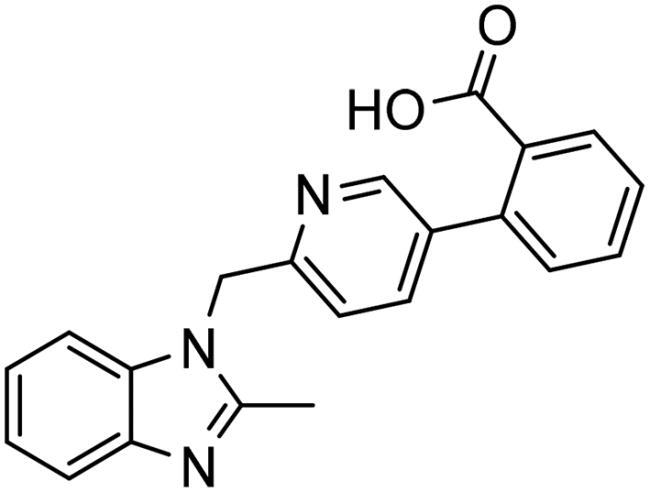 BDE 30700046	**7WT2**	45.6259	−30.9215	−27.7424		96.616	−53.3848	−50.2727	
		**3VW9**	47.0029	−32.8776	−29.4680	342.371	98.732	−47.1233	−43.9454	2.134
		**1QIP**	56.9604	−43.0305	−41.8123		100.261	−25.9745	−24.1925	
**19**	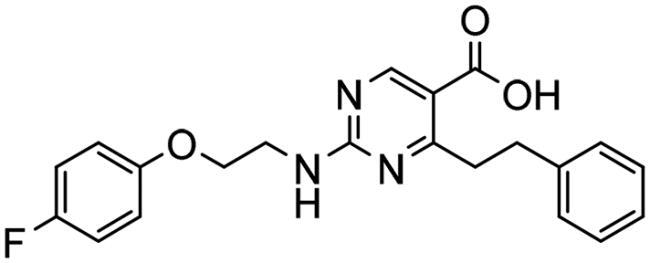 LAS 51175911	**7WT2**	52.1871	−15.6628	−9.8933		119.070	−16.4052	−13.3554	
		**3VW9**	47.4673	−48.0532	−46.7289	380.392	117.354	−51.1520	−44.2896	2.812
		**1QIP**	59.1633	−51.6765	−45.6842		118.981	−53.3030	−48.9750	
**20**	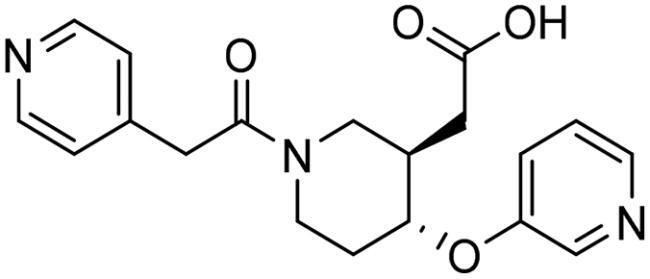 LAS 54560613	**7WT2**	53.5900	−24.3999	−11.8381		107.134	−33.7000	−30.5946	
		**3VW9**	49.9301	−19.8133	−15.0516	354.380	111.150	−46.3165	−32.7633	−1.265
		**1QIP**	57.6918	−58.8787	−53.4532		109.062	−45.8825	−42.2932	
**21**	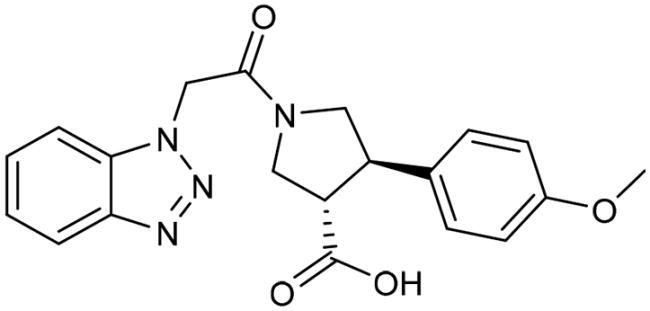 AEM 11794351	**7WT2**	53.3389	−3.9256	−2.3385		117.640	−18.8421	−14.6994	
		**3VW9**	51.0485	−54.4775	−50.4138	379.389	123.091	−47.1134	−36.8170	0.548
		**1QIP**	58.6434	−67.5535	−65.9152		117.463	−41.4131	−37.2623	
**22**	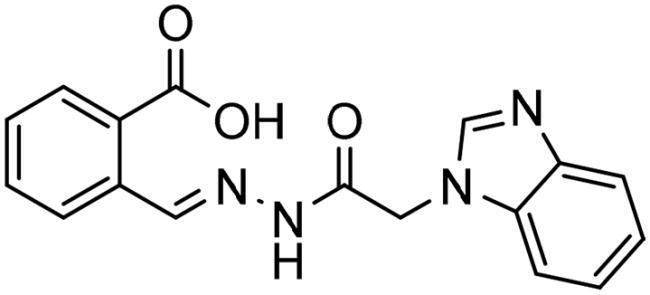 BAS 01905715	**7WT2**	52.4711	−44.1519	−42.3383		107.256	−44.1282	−21.1309	
		**3VW9**	47.9729	−51.0825	−50.1068	321.310	109.514	−33.8452	−16.2046	0.412
		**1QIP**	55.2349	−50.3598	−46.4164		109.145	−30.5987	−27.8368	
**23**	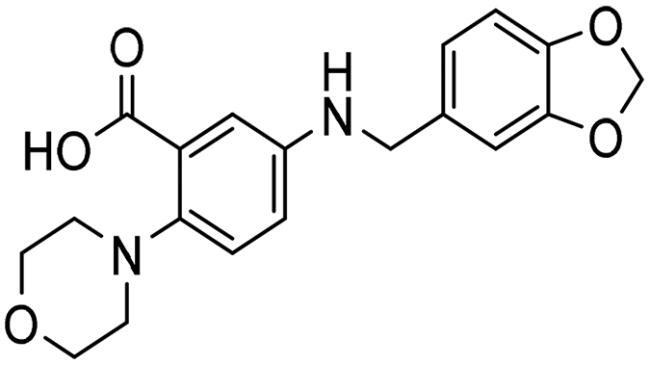 BAS 11358762	**7WT2**	48.0940	−26.4087	−36.6287		104.585	−46.1706	−43.1768	
		**3VW9**	46.3290	−52.1149	−51.2818	356.373	115.064	−54.4708	−50.5476	2.470
		**1QIP**	54.6904	−39.3812	−37.0663		104.838	−43.9506	−38.0854	
**24**	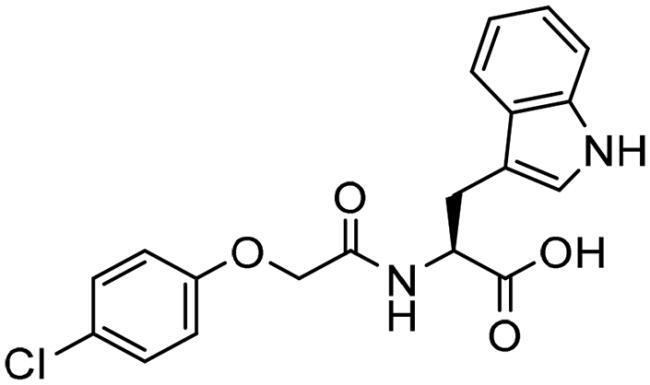 BAS 00556421	**7WT2**	55.1656	−19.5785	−14.9825		111.128	−25.7705	−19.3496	
		**3VW9**	50.6125	−36.5968	−10.6896	371.794	121.459	−49.7227	−48.6497	1.878
		**1QIP**	55.6954	−41.5923	−41.1400		120.543	−43.1483	−41.8783	
**25**	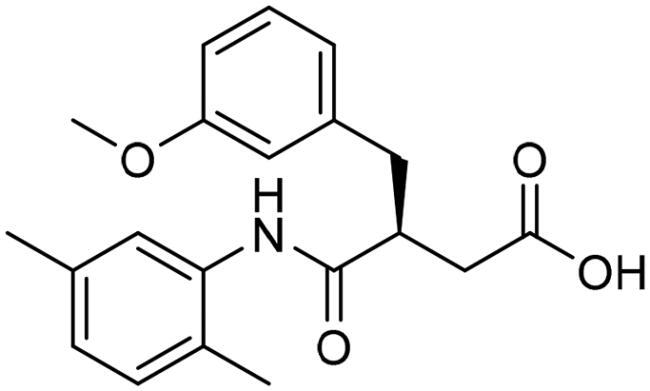 BAS 00341212	**7WT2**	50.3713	−27.0168	−24.2648		104.552	−31.3250	−31.3220	
		**3VW9**	47.8141	−40.1993	−37.0983	340.393	110.088	−48.3627	−39.7898	2.228
		**1QIP**	55.2347	−69.7515	−69.5996		107.044	−44.8387	−40.5774	
**26**	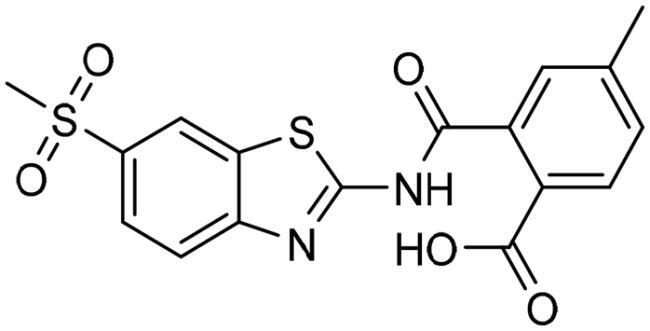 BAS 01177965	**7WT2**	46.6554	−24.8163	1.3823		90.189	−23.4711	−22.6083	
		**3VW9**	48.5603	−26.9008	−24.0772	389.426	97.303	−24.9368	−21.3213	1.408
		**1QIP**	57.0506	−40.1377	−30.8775		101.084	−17.4797	−16.5550	
**27**	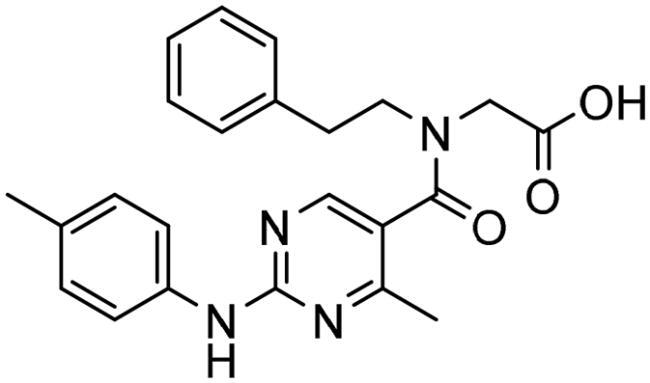 LAS 51154717	**7WT2**	53.9259	−2.2918	−0.6628		118.318	−31.8628	−23.4777	
		**3VW9**	54.1555	−19.6698	−3.7461	403.454	108.072	−50.8928	−44.6046	2.217
		**1QIP**	60.1372	−64.8763	−61.5395		118.154	−61.0124	−49.3263	
**28**	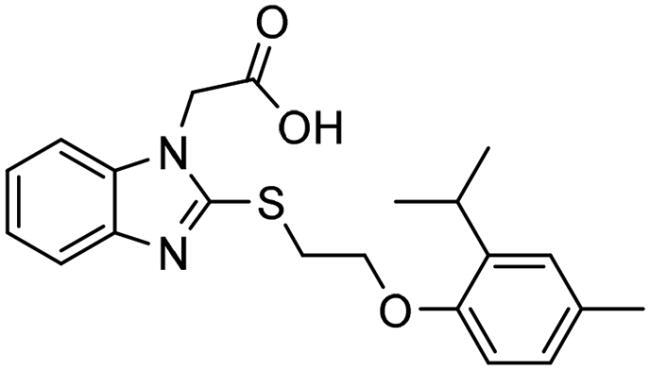 BAS 00450546	**7WT2**	49.0637	−35.6030	−31.6778		103.427	−44.7931	−40.1949	
		**3VW9**	50.4587	−14.1362	−5.1806	383.484	110.734	−54.6542	−51.9360	3.955
		**1QIP**	55.4139	−39.3827	−38.7347		106.751	−53.7478	−48.9591	
**29**	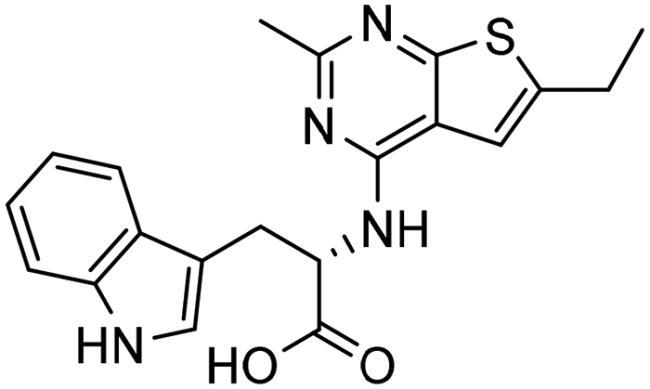 BAS 07396189	**7WT2**	50.4659	−0.3292	−0.2898		104.856	−34.5992	−34.0445	
		**3VW9**	49.4910	−36.4543	−36.4490	379.455	118.399	−41.3994	−41.0815	2.569
		**1QIP**	49.7004	−45.7091	−45.5397		112.536	−33.4419	−33.0965	

### Experimental results

#### Biological evaluation

The *in vitro* inhibitory activities of the 14 selected hits were performed using the human recombinant Glo-I enzyme (rh Glo-I) as previously described. The formation of the Glo-I product, S-D-lactoylglutathione, upon interaction with the substrate is measured spectrophotometrically using a Syringe 2 UV microplate reader by monitoring the increase in absorbance at 240 nm for 200 s. at 25 °C.

The percentage of Glo-I inhibition by the tested compounds was measured by initially determining the absorbance value for each well at 0 and 200 s, and then subtracting the average absorbance value of the blank wells from both the average absorbance values of the enzyme and the compound wells at 0 and 200 s. Afterwards, the percent inhibition was calculated using the following formula:

$$\%\text{inhibition}=(1-\frac{\text{abs}.\text{of the comp}.\text{at}\;200\text{sec}‐\;\text{abs}.\text{of the comp}.\text{at}\;0\;\text{sec}}{\text{abs}.\text{of the enz}.\text{at}\;200\text{sec}‐\;\text{abs}.\text{of the enz}.\text{at}\;0\;\text{sec}})*100\%$$

 The absorbance of the negative control, blank, corresponds to the activity of the enzyme without the interference of any inhibitor. By serial dilution of the hit concentrations from 50 to 0.195 µM, the percent inhibition values for the 14 hits in comparison to the positive control, myricetin, were calculated in three triplicate independent experiments. Subsequently, compounds with more than 60% Glo-I inhibition were regarded as active, whereas those with less than 60% were regarded as weakly active or inactive; therefore, their IC_50_ values were not explored.

However, none of the investigated compounds can be regarded as a lead as the highest percentage of inhibition reportedly obtained was about 18.70% at 50 µM. Compound **19** and compound **28**, whose percentage of inhibitions are 18.70 and 15.80% respectively, can be considered as hits that need further optimisation in order to be converted into lead-like compounds (Table 4).

**Table 4.** Biological screening results of 19 and 28 compounds at 50 µM.

Index	IDNUMBER	Chemical Structure	%Inhibition
**19**	**LAS 51175911**	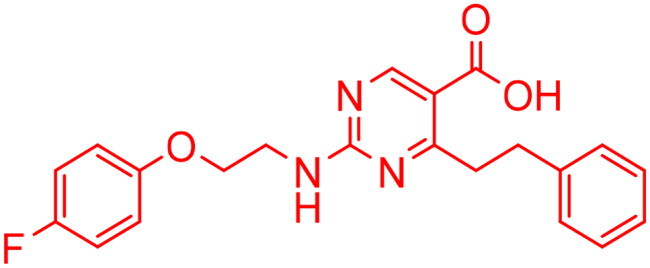	18.70
**28**	**BAS 00450546**	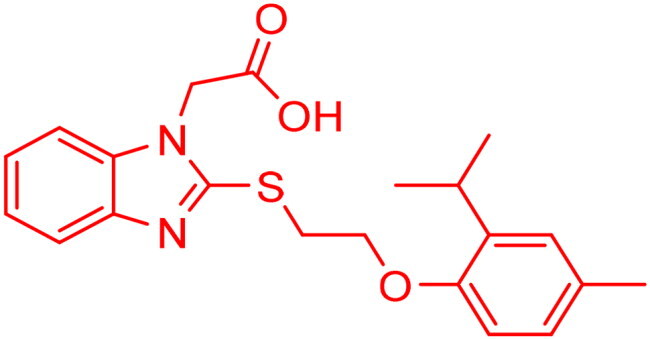	15.80

The inactivity of similar compounds for which binding interaction modes were predicted using a structure-based drug design may be encountered by several potential factors such as inaccuracies and misinterpretations in predicting binding modes, insufficient understanding of target protein structure or dynamics since available structure information fails to capture critical aspects of protein’s conformational changes or binding interactions, and inherent limitations and approximations of computational algorithms and scoring functions for organometallic complexes. It is highly postulated that the limited ability of the carboxylic acid to chelae the zinc atom is the major contributing factor for the compromised activity of compounds 17, 20, 25, and 27. The absence of constructive contact or favourable hydrogen bonds, particularly with amino acids such as Arg37, Arg122, Lys150, and Lys156 at the mouth of the active site, could contribute to further plausible explanation the inactivity in our tested compounds, exemplified by compounds 17, 24, and 28. Furthermore, the inactivity observed in compounds 18 and 25 can be attributed to the humble role played by a small hydrophobic group that occupies the hydrophobic pocket and produces weak hydrophobic interactions. The complexes of the 14 compounds assessed are provided in Supplementary 2.

The percentage of inhibition resulting from compound **19** could be attributed to the ability of the ionisable carboxylic acid at physiological pH to form a bidentate coordination with the zinc metal and the phenyl ring to occupy the hydrophobic pocket (Figure 7A). Despite being relatively small and showing some % inhibition, this compound suggests the presence of extra rooms relative to the binding site that can be exploited as a starting point to derive more potent Glo-I inhibitors.

#### Flexible docking of the most active compounds

In order to obtain more information concerning the binding pattern of the 2 most active compounds, compounds 19 and 28 were further docked using the computationally intensive Flexible Docking protocol. This protocol involves the following sequential steps: generation of receptor conformations using ChiFlex; creation of ligand conformations; execution of ligand docking into each active protein conformation site using LibDock; clustering to eliminate similar ligand poses; refinement of selected protein side chains in the presence of the rigid ligand using ChiRotor; and finally, culminating in a last ligand refinement using CDocker.

**Figure 8.**
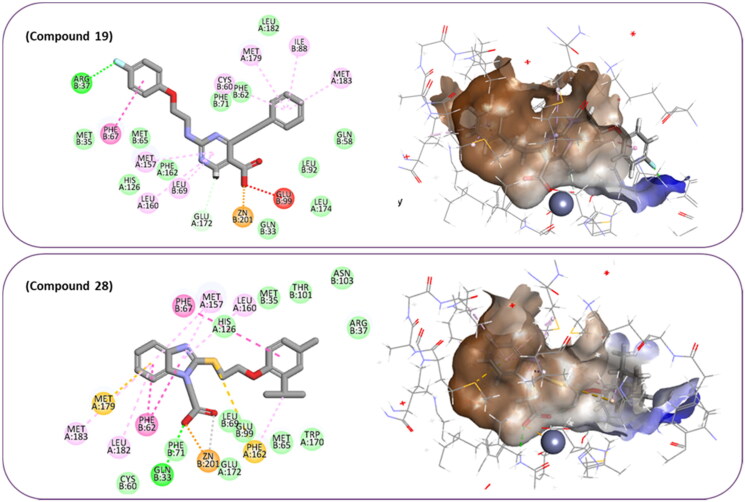
The docked pose of compound number **19** and compound number **28**, in the active site of Glo-I PBD code 7WT2 along with a 2D interaction map using Flexible Docking protocol. The binding site is represented as a hydrophobic surface, and the Zinc metal as a grey sphere.

For the purpose of exhibiting the desired activity, varying the distance between the carboxylic acid and the phenyl ring gives the carboxylic acid the opportunity to manoeuvre freely within the active site with the hope to adopt better orientation while chelating zinc atom at the active site. Another modification in compound 19 involves replacing the relatively weak hydrogen bond acceptor, fluorine, with better hydrogen bond acceptor, such as a hydroxy group which is expected to perform an ion diploe interaction with Arg37. Additionally, in compound 28, the incorporation of a strong hydrogen bond acceptor into the phenyl ring of 2-isopropyl-4-methylphenoxy group aims to foster stronger interactions with the positively charged mouth of the Glo-I active site (Figure 8).

## Materials and methods

### Computational materials

Sketching the fragments, evoluted and purchased compounds was performed using ChemBioDraw Professional 15.0 (PerkinElmer Inc., MA). Preparation of the Glo-I enzyme was performed using Discovery Studio (DS) 2022 from BIOVIA® (formerly Accelrys®) Software Inc. (San Diego, CA, USA). The ASINEX® fragments library was used in a FBDD strategy to develop novel potential hits, and ASINEX® screening collections database was virtually screened for potential hits. Presentation quality images were generated using DS. GraphPad Prism 8 (GraphPad Software Inc., CA) was used in calculation and plotting of the % enzyme inhibition values.

### Experimental materials

The human recombinant Glo-I (rh GLO-I) enzyme from (R&D Systems® Corporation, USA) was used to assess the inhibitory activity of Glo-I via an *in vitro* assay. The final selected compounds obtained from virtual screening of ASINEX® database were purchased from ASINEX® (Amsterdam, The Netherlands, EU) research area. All solvents and reagents, including a mono and Di-phosphate buffer, deionised water, DMSO, glutathione, and methylglyoxal was provided from Acros (Thermo Fisher Scientific, NJ, USA) and Sigma-Aldrich Co. with the highest purity via local vendors. The biological activities of the purchased compounds were performed *in vitro* using UV Synergi 2 microplate reader.

### Computational methods

There are customarily three basic steps evolved into a very successful computational approach of FBDD which are: designing a good diverse fragment library as the initial step; computational docking or screening of the designed library as the second step; and computational fragment optimisation methods for growing, linking, or direct connection (joining or merging) the fragments to generate potential lead compounds as the third step.

#### Preparation of the Glyoxalase-I enzyme

The three different crystal structure models of the Glo-I enzyme were constructed for further preprocessing using Discovery studio (DS) 2022 from Biovia® software Inc, where the baseline coordinates for the investigated systems were retrieved from PDB. Seven Glo-I X-ray crystal structures were available in the protein data bank with entry codes 7WT2, 3VW9, 3W0T, 3W0U, 1QIN, 1QIP, and 1FRO. In this study, three crystal structures were utilised as working models with PDB codes 7WT2, which correspond to human Glo-I in complex with TLSC702 reveal inhibitor (5ZO) at 2.0 Å resolution; 3VW9, which correspond to human Glo-I in complex with N-hydroxypyridone derivative inhibitor (HPJ) at 1.47 Å resolution; 1QIP, which correspond to human Glo-I in complex with S-P-nitrobenzyloxycarbonyl glutathione inhibitor (GNB) at 1.72 Å resolution.

The PDB files were checked for missing loops, incomplete residues, and alternative conformations using Protein Report tool within DS. Then, the Glo-I crystal structures were prepared using Prepare Protein protocol to protonate the residue at a pH equal to 7.4, standardise the atom’s names, remove all of the missing residues and any additional conformations that might have been stimulated, and fixing the connectivity and order of the bonds. The prepared complexes were first solvated by immersing them in a cubic box of pre-equilibrated explicit water, whose minimum distance from the boundary is 7.0 Å, using the Solvate protocol. Then, the solvated systems were minimised in three sequential steps by applying CHARMm forcefield with Smart Minimiser Algorithm using the Minimisation protocol. In the first step of minimisation, the heavy atoms of the backbone and side chains were exposed to a harmonic restraint. Subsequently, only the backbone atoms were restrained, and finally the entire system was allowed to move and adjust their position.

#### Fragment library design

In the interest of designing a good fragment library, the ASINEX fragments library, which is composed of 20061 structurally diverse fragments, was utilised in this research. The library was downloaded as a sdf file format, imported into DS software, and then filtered to aid the selection of the fragments comprising a library based on a set of rules, known as the “Rule of Three”, with the exception of one criterion, which is the hydrogen bond acceptor. The filtration was performed by calculating the 2D descriptors of fragments using Calculate Molecular Properties protocol within DS.

Then, the selected fragments were converted into a 3D database using the Build 3D Database protocol, which uses the Catalyst algorithm^35^. The created 3D database is automatically indexed with sub-structure, pharmacophore features, and shape information to facilitate fast database screening. The protocol was utilised with default parameters except for Conformation Method, which was set to Best.

#### Primary screening of the fragment library

Once a fragment library has been built, it was followed by a virtual screening step to identify potential hit fragments using a pharmacophore-based screening step prior to fragment docking. A customised pharmacophoric zinc binding feature (ZBF) was utilised as a search query using the Search 3D Database protocol at default parameters except for the Search Method, which was set to Best, to identify fragments that possess zinc binding groups. The customised ZBF was generated using the Customise Pharmacophore Features within DS and then Add Features from the Dictionary. Then, the mapping fragments were collected for molecular docking.

#### Fragment docking and scoring

The Glo-I active site of the three distinct crystal structures was defined using the Define and Edit Binding Site tool into the zinc-hydrophobic pocket region prior to the fragment docking. The zinc-hydrophobic pocket region with a 7.0 Å sphere radius was defined in the crystal structures of 7WT2 and 3VW9, while a larger 7.3 Å radius sphere was defined in the crystal structure of 1QIP. Then, the CHARMm-based MCSS docking protocol was employed for the retained fragments in each of three investigated systems with default parameters except for Iteration Profile, which was set to Conjugate^27^.

#### Calculation of the total binding energy and ligand efficiency of docked fragments

The total binding energy (TBE) of the top-ranked docked fragments in the Zn-hydrophobic sphere, which was determined by a threshold cut-off, was calculated using the In Situ Ligand Minimisation and then the Calculate Binding Energies protocols^28^. The In Situ Ligand Minimisation protocol was conducted using the Adopted Basis Newton-Raphson (NR) algorithm. The Calculate Binding Energies protocol was performed at default parameters with the exception of the Ligand Conformation Entropy, which was set to True, and the Implicit Solvent System, for which the Poisson Boltzmann with non-polar surface area (MM-PBSA) model was chosen^29^. Then, by employing the Calculate Ligand Efficiency protocol and the fragments with their binding energies as input ligand, ligand efficiency was calculated for the top-ranked fragments in terms of binding energy.

#### Fragment evolution

There are different techniques for designing large, potent lead compounds from small initial fragment hits that have sufficiently high ligand efficiency and low binding affinity, including growing, linking, replacing, and merging/joining fragments. In this research, the selected fragments were evoluted in three complex systems using the De Novo Evolution protocol, which is a fragment growing strategy, in both Full and Combinatorial Evolution Modes. The De Novo Library Generation protocol, particularly the link library type was employed to create a fragment library in text or binary format to be used later in the *de novo* protocols.

Before Full Evolution Mode was applied to the selected fragments, the Define and Edit Binding Site tool was used to define the Glo-I binding site for each complex system using a sphere that covered all significant amino acid residues. The cavity that houses the bound ligand was enclosed by the sphere. All residues in the binding site that might be relevant to ligand binding were included by expanding the sphere to 10 Å in each of three Glo-I systems. Then, the De Novo Evolution protocol was applied to the selected fragments with a Maximum of 20 Population Size per fragment, a Maximum of one Number of Generations, the Best Search Method, and with the Rejection of carboxylic acid groups within the Fragment Filters, while all other parameters were kept as default. Whereas the De Novo Evolution protocol was applied in Combinatorial Evolution Mode to the selected fragments with 7.0 Å sphere model created at each link point, a Maximum Alignment Angle of 20° Link Points, and with the Best Search Method, while all other parameters were kept as default.

#### Molecular docking and calculation of the TBE of evoluted compounds

Prior to molecular docking, the evoluted compounds were prepared using Prepare Ligands protocol using default parameter values with the exception of the Ionisation Method, which was set to Rule Based, the Generate Tautomers in which the Enumerate What was set to Canonical, and the Generate Isomers which was set to False.

Two molecular docking algorithms within DS, CDocker and LibDock, were used to dock the retained evoluted compounds acquired from each complex system into its defined Glo-I active site to assess active site binding in the same way and with the essential geometry as observed with each of the three complex systems. The same sphere model generated in Glo-I enzyme structure for the De Novo Evolution protocol was utilised for docking objectives by both CDocker and LibDock protocols. Furthermore, each complex system received its own docking of the entirety of the evoluted compounds collected from all complex systems.

The CDocker protocol was implemented using default parameter values except for Using Full Potential which was set to true. While the LibDock protocol was implemented using default parameters with the exception of the Conformation Method which was set to Best, the Energy Threshold in Conformation Method which was reduced to 10 kcal/mol, and the Minimisation Algorithm which was set to Smart Minimiser. Afterwards, similar to the approach was used in calculating the binding energies of the docked fragments, the output docked poses of the evoluted compounds from both CDocker and LibDock protocols were subjected to energy minimisation using the In Situ Minimisation protocol. Then, the binding energies of the minimised ligands were calculated using the Calculate Binding Energies protocols.

#### Virtual screening of commercial database

The ASINEX® screening collections database was virtually screened using a variety of strategies to identify similar compounds for potential evoluted hits including, ligand 3D pharmacophore mapping for compounds containing a carboxylic acid group as a zinc binding feature, a guided pharmacophore model for each potential evoluted hits, ligand 3D pharmacophore mapping of each resulting compounds obtained from its guided pharmacophore model, and finally a 2D similarity searching using 2D structural fingerprints.

A carboxylic acid group was the only 3D pharmacophore query of zinc binding feature that was specified for the Ligand Pharmacophore Mapping technique from the outset. The protocol was applied at default parameters with Best conformation method. A guided ligand-based pharmacophore model was built utilising structural information for each evoluted potential hit. Then, it was used as a query in virtual screening of the resulting compounds that included a carboxylic acid group using a Ligand Pharmacophore Mapping protocol. The protocol was applied at default parameter with Best conformation and Flexible Sitting methods. Finally, 2D descriptors were used to encode the presence of 2D substructural fingerprints in a compound in order to find compounds similar to the reference ligands, which are the resulting compounds obtained from each guided pharmacophore model. This process was performed using Find Similar Molecules by Fingerprints protocol with the Tanimoto Similarity Coefficient and FCFC-4 as a Predefined Set of fingerprints. However, our selection of similar compounds was guided by the final evoluted compounds.

#### Molecular docking and scoring of similar compounds

Similar compounds were prepared, molecular docked, and scored in a manner similar to that used for the evoluted compounds. Further inspection and filtration were conducted to select the most suitable similar compounds, which will be selected for calculating the binding energy protocol.

#### Selection of the final compounds

In order to identify the most definitive potential inhibitors of the Glo-I enzyme, further visual inspection and filtration were performed. Then, the retained hits were subjected to the Cluster Ligand protocol to select the final 14 acquired compounds and to experimentally evaluate their inhibitory activities. The protocol was applied at default parameters with 14 Numbers of Clusters and FCFP-4 as a Predefined Set of properties. Synthetic strategies of the 2 most active compounds can be found in supplementary 3.

### Experimental methods

#### In vitro enzyme assay

Following the manufacturer’s procedure (R&D Systems®, Inc., Minneapolis, MN, USA), the *in vitro* biological activities of the selected compounds were conducted by measuring their inhibitory activities against human recombinant Glo-I. In our laboratory, the enzyme was reconstituted by dissolving it to a concentration of 0.5 mg/mL in sterile deionised water, freezing at −79 °C, and then thawing it on the day of testing. Absorbances were measured using a double-beam UV–Vis spectrophotometer at a maximum wavelength of 240 nm for 200 s at 25 °C after dissolving the test compounds in dimethyl sulfoxide to make a 10 mM stock solution. The buffer solution was prepared using 0.5 M sodium phosphate dibasic stock solution and 0.5 M sodium phosphate monobasic stock solution. The substrate solution, containing 706 μL of freshly prepared glutathione and 706 μL of freshly prepared MG, was added to 25 ml of assay buffer and the obtained mixture was allowed to incubate in water bath at 37 °C for 15 min. The enzyme was prepared by mixing 17.75 μl of it with a 10 ml incubated buffer to be used later in the enzyme activity wells at a volume of 49 μl, plus to 150 μl buffer and 1 μl DMSO. The blank test was 10 ml of incubating buffer to be used later in the blank activity wells at a volume of 49 μl, plus to 150 μl buffer and 1 μl DMSO. The tested compounds were prepared by serially diluting them with DMSO to be used later in the compound activity wells at a volume of 1 μl, plus to 150 μl buffer and 49 μl enzyme. All tests were performed through three independent experiments to be conducted in triplicate. Then, the inhibitory activities assessed were followed by measuring their IC_50_ values using GraphPad Prism 8. Myricetin was used as a positive control with an IC_50_ equals to 3.38 ± 0.41 (µM).

#### Flexible docking of the most active compounds

The same docking sphere used previously in this work which was generated in the Glo-I enzyme structure (3VW9) was utilised for docking using the Flexible Docking protocol within DS. All parameter values in the protocol were set to default, with the exception of the Conformation Method in 'Generate Ligand Conformation,' which was set to Best. The flexible group that will be permitted to move throughout the docking process is defined as amino acids that correspond to the Glo-I active site: Gln33B, Glu172A, Glu99B, His126A, Phe162A, Met65B, Met183A, Met179A, Met157A, Leu69B, and Cys60B.

## Conclusion

A fragment-based drug design strategy had been implemented for identifying potential Glo-I inhibitors. The resulting compounds were served as a benchmark for choosing similar compounds from the ASINEX^®^ commercial database. Being a FBDD technique is highly dependent on the quality of the protein structure, three crystal structures of Glo-I were utilised. Subsequently, 14 compounds were selected as potential candidates for biological evaluation. The screened compounds displayed weak activity, with Glo-I inhibition percentages ranging from 0 to 18.70%. Nevertheless, compounds **19** and **28** were identified as promising hits, that upon further optimisation could be converted into lead-like compounds. Thus, the identified hits serve as a starting point for further optimisation in attempts to identify potent Glo-I inhibitors. Collectively, through the application of this method, we are able to identify compounds that have a higher possibility of success and impact while gaining important insights into the potential advantages and disadvantages of synthesising evoluted compounds. This also allows us to explore strategies for modifying the structure of the evoluted compounds to improve the likelihood of exhibiting the desired activities and support the efficiency of FBDD approach. Additionally, the identified similar hits would be optimised in an effort to boost their prospective activities in order to produce a lead like compound with potent inhibitory activity.

## Acknowledgements

This study was funded by the Deanship of Research at the Jordan University of Science and Technology (grant number 131–2021 & 175–2023)

## Supplementary Material

Supplemental Material
