# Supplementary material for: Computational fragment-based drug design of potential Glo-I inhibitors
Source: J Enzyme Inhib Med Chem. 2024 Jan 22;39(1):2301758. doi: 10.1080/14756366.2024.2301758 (PMC10810659; doi:10.1080/14756366.2024.2301758)
Supplement: Supplemental Material [file IENZ_A_2301758_SM5148.zip › supplementary 1.pdf]

Supplementary 1: Left panel: Different guided pharmacophore models generated from evoluted compounds. Middle panel: The compounds have been eliminated for clarification. The feature types in the pharmacophores are: HBA (green), NI (dark blue), HY (sky blue), and RA (brown). Right panel: Vaildation.

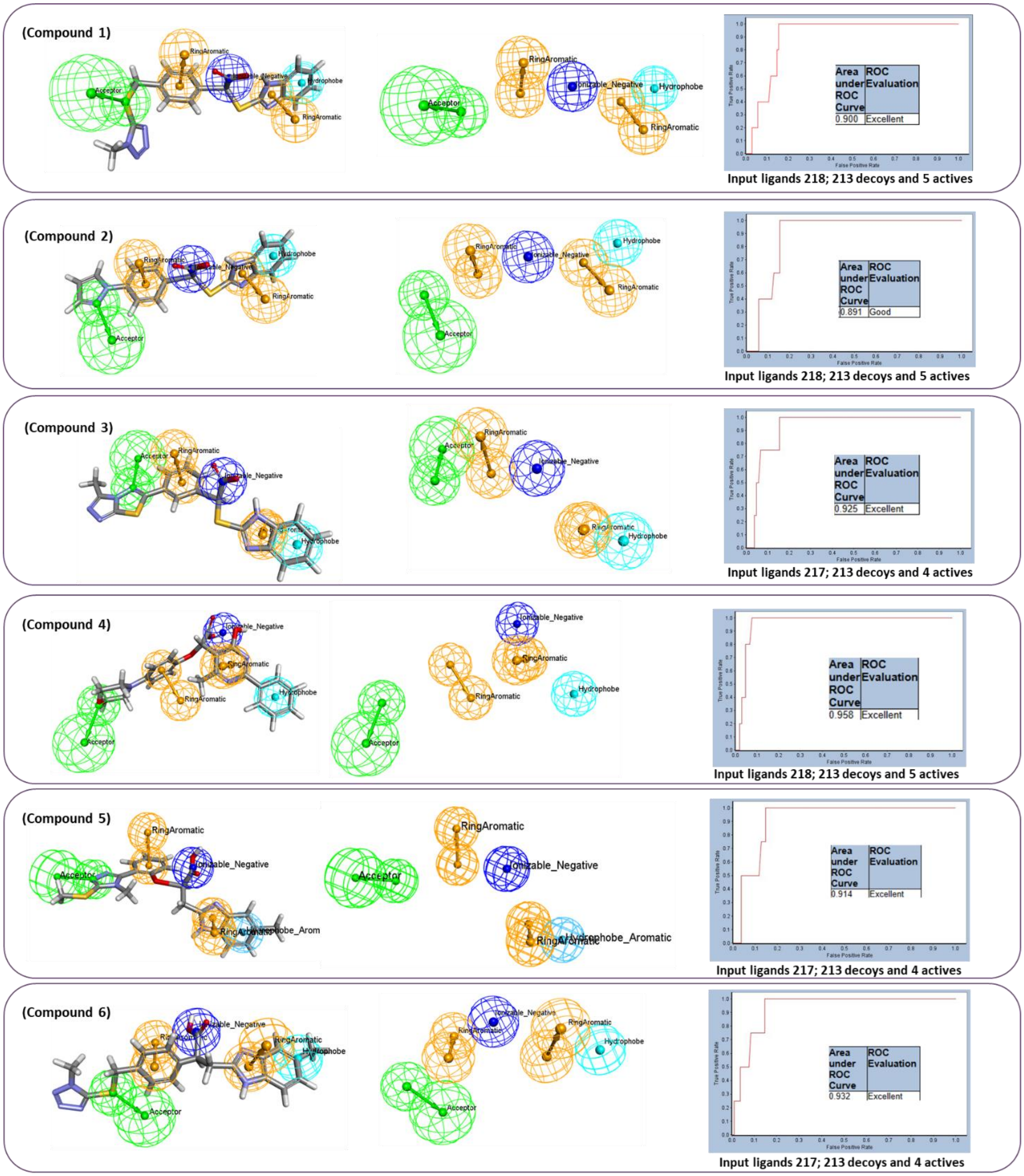

(Compound 7)

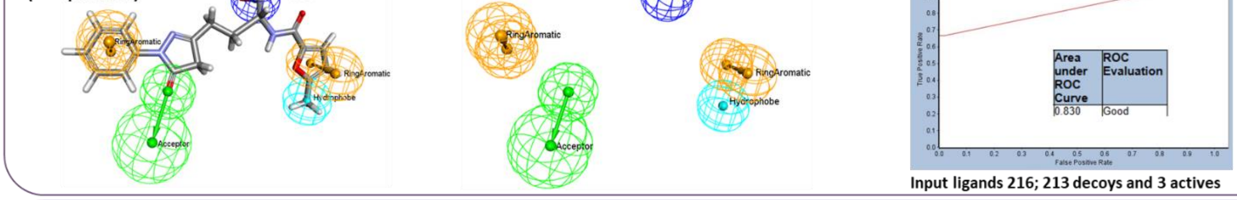

Input ligands 216; 213 decoys and 3 actives

(Compound 8)

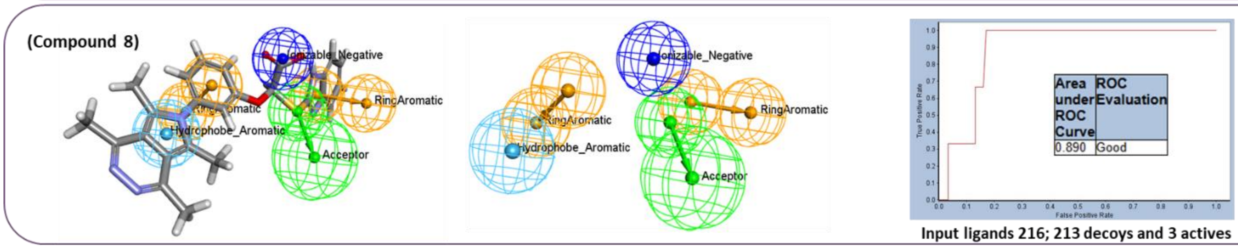

Input ligands 216; 213 decoys and 3 actives

(Compound 9)

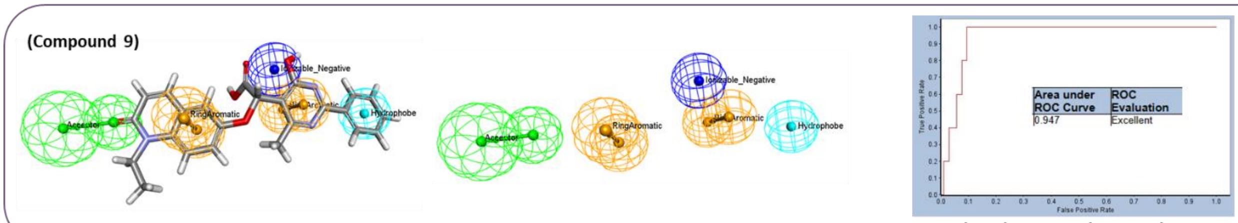

Input ligands 218; 213 decoys and 5 actives

(Compound 10)

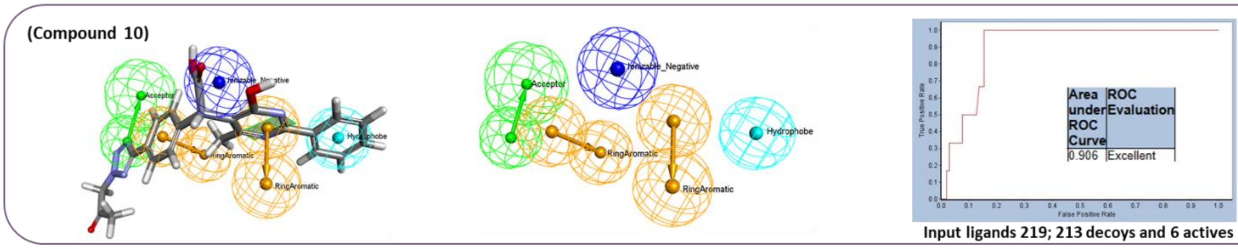

Input ligands 219; 213 decoys and 6 actives

(Compound 11)

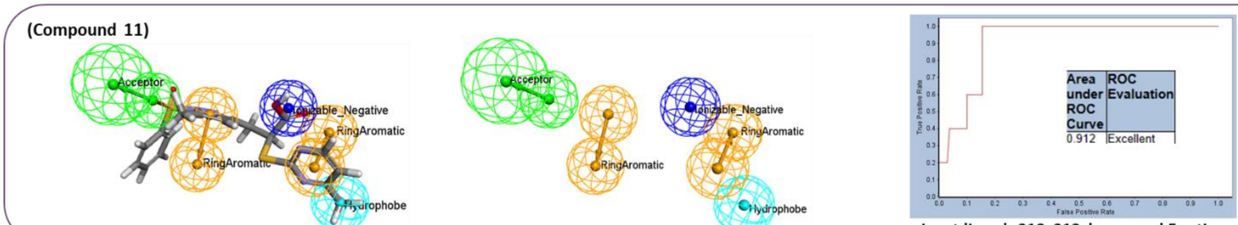

Input ligands 218; 213 decoys and 5 actives

(Compound 12)

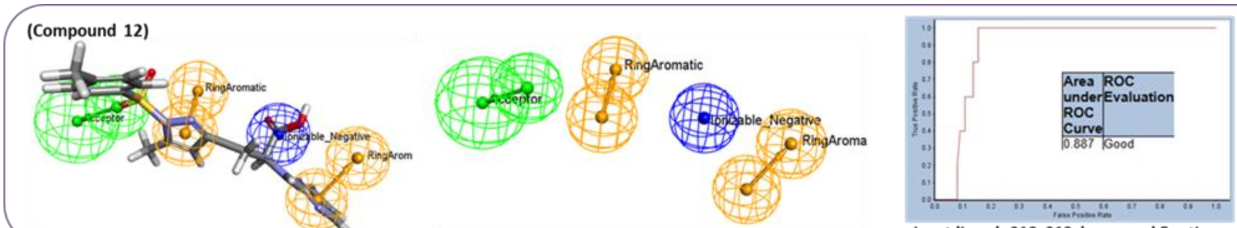

Input ligands 218; 213 decoys and 5 actives

(Compound 13)

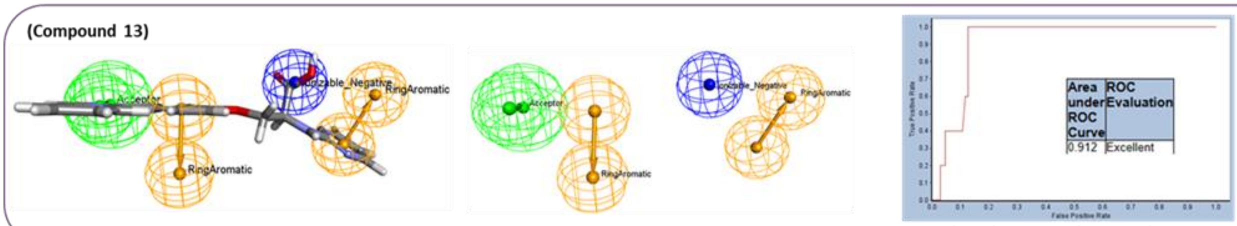

Input ligands 218; 213 decoys and 5 actives

(Compound 14)

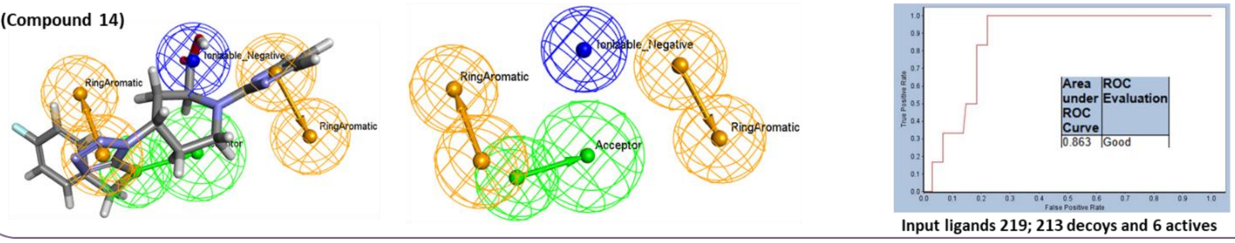

Input ligands 219; 213 decoys and 6 actives

(Compound 15)

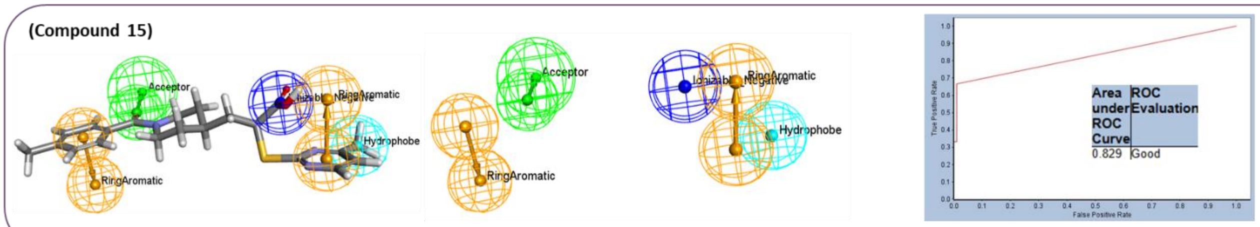

Input ligands 216; 213 decoys and 3 actives
