# Supplementary material for: Computational fragment-based drug design of potential Glo-I inhibitors
Source: J Enzyme Inhib Med Chem. 2024 Jan 22;39(1):2301758. doi: 10.1080/14756366.2024.2301758 (PMC10810659; doi:10.1080/14756366.2024.2301758)
Supplement: Supplemental Material [file IENZ_A_2301758_SM5148.zip › supplementary 2.pdf]

Supplementary 2: The docked poses of 14 compounds tested in the active site of Glo-I PBD code (3VW9). The binding site is represented as a hydrophobic surface, and the Zinc metal as a grey sphere

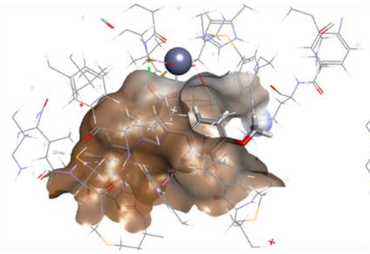

(Compound 16)

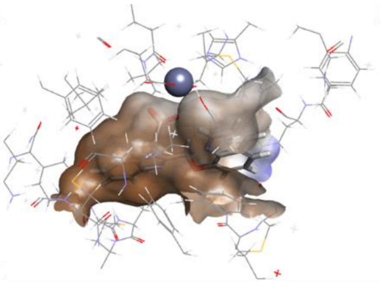

(Compound 17)

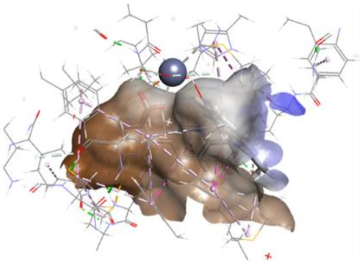

(Compound 18)

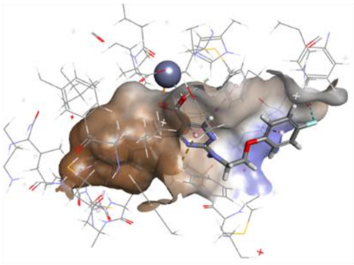

(Compound 19)

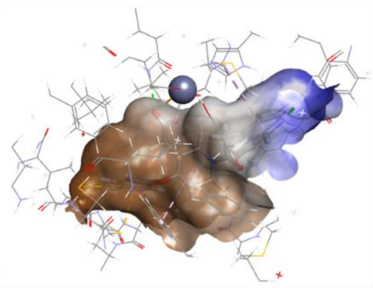

(Compound 20)

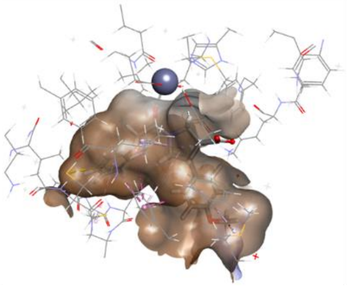

(Compound 21)

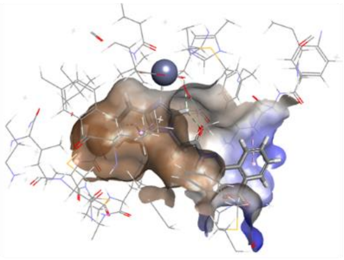

(Compound 22)

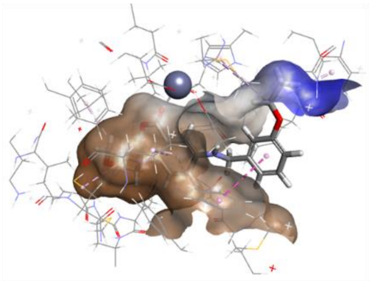

(Compound 23)

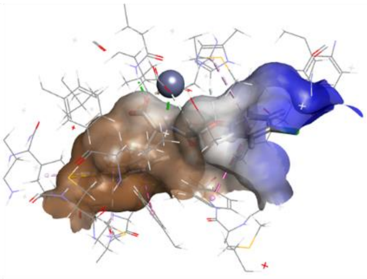

(Compound 24)

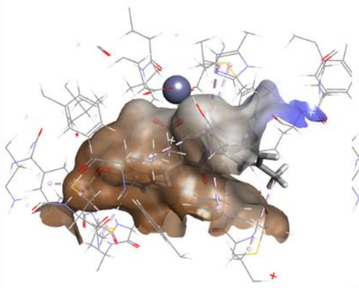

(Compound 25)

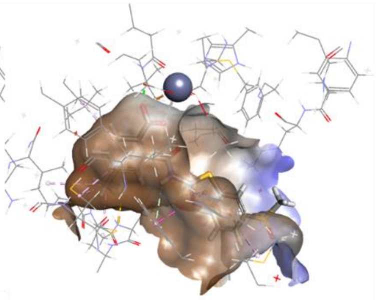

(Compound 26)

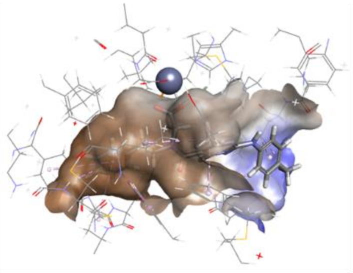

(Compound 27)

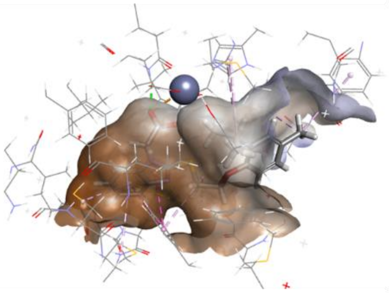

(Compound 28)

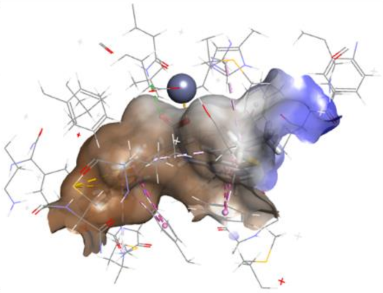

(Compound 29)
