# Supplementary figures and images for: Computational fragment-based drug design of potential Glo-I inhibitors
Source: J Enzyme Inhib Med Chem. 2024 Jan 22;39(1):2301758. doi: 10.1080/14756366.2024.2301758 (PMC10810659; doi:10.1080/14756366.2024.2301758)

### Supplementary 3: Synthetic strategies of the 2 most active compounds

#### Compound 28

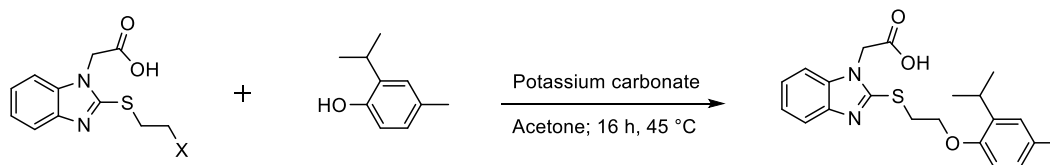

#### Compound 19

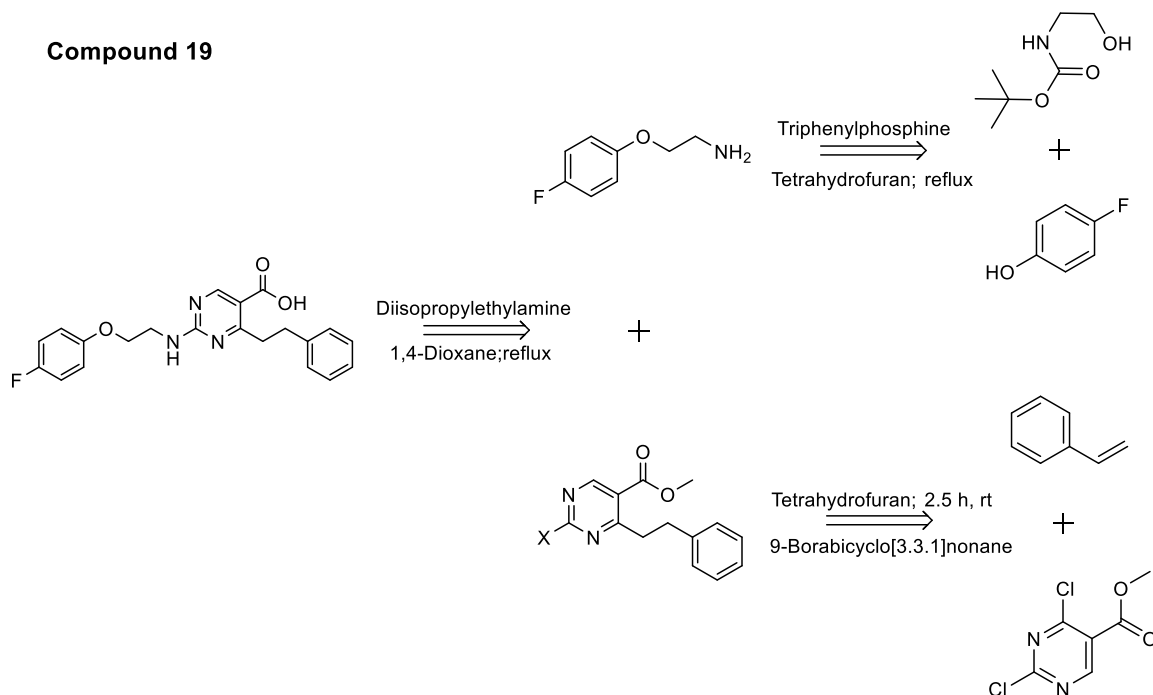

Supplement: Supplemental Material [file IENZ_A_2301758_SM5148.zip › Supplementary 3.pdf]
